# Persistence of post-stress blood pressure elevation requires activation of astrocytes

**DOI:** 10.1038/s41598-024-73345-4

**Published:** 2024-10-03

**Authors:** Yohei Hasebe, Shigefumi Yokota, Isato Fukushi, Kotaro Takeda, Masashi Yoshizawa, Hiroshi Onimaru, Yosuke Kono, Shuei Sugama, Makoto Uchiyama, Keiichi Koizumi, Jouji Horiuchi, Yoshihiko Kakinuma, Mieczyslaw Pokorski, Takako Toda, Masahiko Izumizaki, Yasuo Mori, Kanji Sugita, Yasumasa Okada

**Affiliations:** 1 Department of Pediatrics, School of Medicine, University of Yamanashi, Chuo, Yamanashi Japan; 2 Clinical Research Center, Murayama Medical Center, 2-37-1 Gakuen, Musashimurayama, Tokyo, 208-0011 Japan; 3 Department of Anatomy and Morphological Neuroscience, Shimane University School of Medicine, Izumo, Shimane Japan; 4 Graduate School of Health Sciences, Aomori University of Health and Welfare, Aomori, Japan; 5 Faculty of Rehabilitation, School of Health Sciences, Fujita Health University, Toyoake, Aichi Japan; 6 Department of Physiology, Showa University, School of Medicine, Tokyo, Japan; 7 Center for Medical Sciences, International University of Health and Welfare, Otawara, Tochigi Japan; 8 Department of Synthetic Chemistry and Biological Chemistry Graduate School of Engineering, Kyoto University, Kyoto, Japan; 9 Department of Biomedical Engineering, Graduate School of Science and Engineering, Toyo University, Saitama, Japan; 10 Department of Physiology, Nippon Medical School, Tokyo, Japan; 11 Institute of Health Sciences, University of Opole, Opole, Poland

**Keywords:** Cell biology, Neuroscience, Physiology

## Abstract

The reflexive excitation of the sympathetic nervous system in response to psychological stress leads to elevated blood pressure, a condition that persists even after the stress has been alleviated. This sustained increase in blood pressure, which may contribute to the pathophysiology of hypertension, could be linked to neural plasticity in sympathetic nervous activity. Given the critical role of astrocytes in various forms of neural plasticity, we investigated their involvement in maintaining elevated blood pressure during the post-stress phase. Specifically, we examined the effects of arundic acid, an astrocytic inhibitor, on blood pressure and heart rate responses to air-jet stress. First, we confirmed that the inhibitory effect of arundic acid is specific to astrocytes. Using c-Fos immunohistology, we then observed that psychological stress activates neurons in cardiovascular brain regions, and that this stress-induced neuronal activation was suppressed by arundic acid pre-treatment in rats. By evaluating astrocytic process thickness, we also confirmed that astrocytes in the cardiovascular brain regions were activated by stress, and this activation was blocked by arundic acid pre-treatment. Next, we conducted blood pressure measurements on unanesthetized, unrestrained rats. Air-jet stress elevated blood pressure, which remained high for a significant period during the post-stress phase. However, pre-treatment with arundic acid, which inhibited astrocytic activation, suppressed stress-induced blood pressure elevation both during and after stress. In contrast, arundic acid had no significant impact on heart rate. These findings suggest that both neurons and astrocytes play integral roles in stress-induced blood pressure elevation and its persistence after stress, offering new insights into the pathophysiological mechanisms underlying hypertension.

**Supplementary Information:**

The online version contains supplementary material available at 10.1038/s41598-024-73345-4.

## Introduction

Hypertension is a major public health concern. Although the exact pathogenesis of hypertension remains unclear in most cases, it is thought to be at least partially caused by persistently elevated sympathetic nervous activity^1^. Blood pressure is elevated in response to psychological stress, which activates the sympathetic nervous system in both animals and humans. However, once elevated blood pressure does not immediately return to pre-stress resting levels even after the stress is relieved^2,3^. Therefore, understanding the mechanisms behind this sustained post-stress blood pressure elevation could provide valuable insights into the pathophysiology of hypertension^4,5^.

The prolonged activation of sympathetic neural activity may represent a form of neural plasticity^6,7^. Recent studies have shown that not only neurons, but also glial cells, particularly astrocytes, play critical roles in various forms of neural plasticity^8–12^. For example, astrocytes are essential for the persistence of pain^13,14^, learning of motor skills^15^, formation of memory^16^ and post-hypoxic persistent respiratory augmentation^12^. Based on these findings, we hypothesized that astrocytes are involved in the persistence of post-stress blood pressure elevation.

In this study, we aimed to test this hypothesis using arundic acid, a widely used inhibitor of astrocyte function^12,17–22^. First, we examined the cell-type specificity (neuronal and astrocytic) of arundic acid’s effects to confirm that its inhibitory action targets astrocytes. Next, we subjected rats to psychological stress and conducted histological analyses to determine whether cells in cardiovascular brain regions were activated, and whether arundic acid pre-treatment inhibited this stress-induced activation. Finally, we assessed the effects of arundic acid pre-treatment on blood pressure and heart rate (HR) responses during and after psychological stress in unanesthetized, unrestrained rats.

## Results

### Cell-type specificity of arundic acid action in cultured astrocytes and neurons

**Fig. 1:**
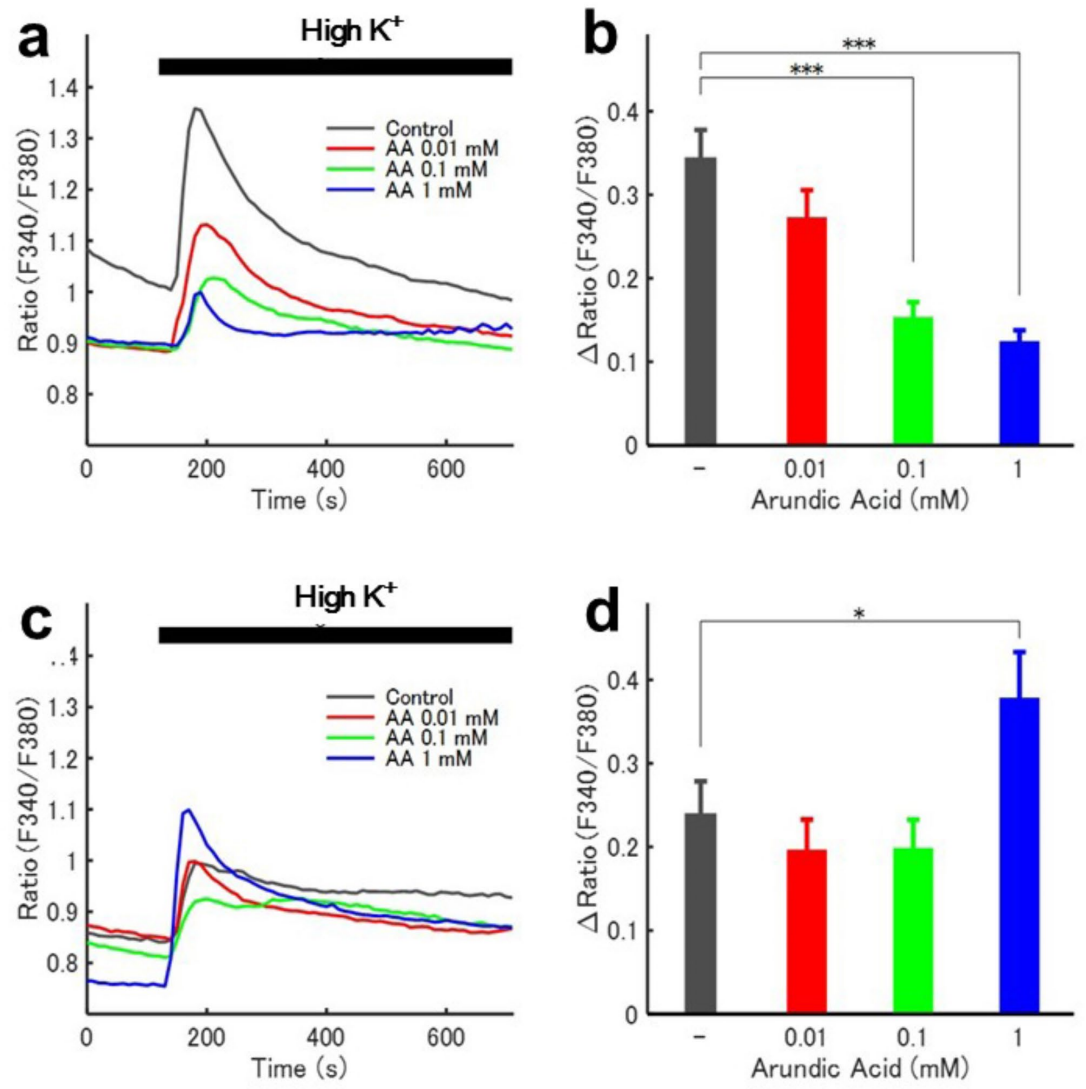
Cell-type specificity of arundic acid actions analyzed by ratiometric calcium imaging. (**a**,**b**) Arundic acid at concentrations of 0.01, 0.1 and 1 mM inhibited high potassium-induced astrocytic activation in a dose-dependent manner. (**c**,**d**) In contrast to astrocytes, arundic acid at concentrations of 0.01 or 0.1 mM did not affect high potassium-induced neuronal activation, while a higher concentration (1 mM) of arundic acid paradoxically activated neurons.

In vehicle-treated cultured astrocytes, high potassium stimulation caused a rapid elevation in ratio (F340/F380) as observed through ratiometric calcium imaging, followed by a slow spontaneous reduction, indicating that astrocytes were activated by the high potassium stimulus (Fig. 1a). Arundic acid at concentrations of 0.01, 0.1 and 1 mM inhibited high potassium-induced astrocytic activation in a dose-dependent manner (Fig. 1a,b).

In cultured neurons treated with vehicle, high potassium stimulation similarly induced a rapid elevation in ratio (F340/F380), which remained persistently high, indicating neuronal activation (Fig. 1c). However, in contrast to astrocytes, arundic acid at concentrations of 0.01 or 0.1 mM did not affect high potassium-induced neuronal activation, and a high concentration (1 mM) of arundic acid paradoxically activated neurons (Fig. 1c,d). These findings confirm that the inhibitory effect of arundic acid is specific to astrocytes, as it does not suppress neuronal activation and may even stimulate neurons at particularly high concentrations.

### Cell-type specificity of arundic acid action in acute brain slices

We examined the effects of arundic acid (0.1-1 mM) on cellular responses to high potassium (40 mM KCl) using calcium imaging in acute brain slices. Figure S1 illustrates a typical example of the effect of arundic acid (0.1 mM). The imaged cells were classified into two groups: putative astrocytes (No. 1–12, marked in yellow in Fig. S1a) and putative neurons (No. 1–6, marked in light blue in Fig. S1a). In the control condition (without arundic acid pre-treatment), putative astrocytes displayed a fluorescence intensity pattern characterized by a spike-like increase in response to high potassium (Fig. S1b,b’), while putative neurons exhibited a slow, plateau-like increase in fluorescence intensity (Fig. S1d,d’). After 30 min of pre-treatment with 0.1 mM arundic acid, the spike-like increases in calcium signals disappeared in the astrocytes, while the plateau-like increases persisted (Fig. S1c,c’). The responses of putative neurons to high potassium remained similar to the control condition (Fig. S1e,e’).

### Histochemical analyses

We evaluated the effects of air-jet stress loading, both with and without arundic acid pre-treatment, on cellular activation in cardiovascular and non-cardiovascular brain regions using c-Fos immunohistology. To identify the types of cells expressing c-Fos, we conducted dual staining for: (a) c-Fos and the neuron-specific marker, neuronal nuclear antigen (NeuN), (b) c-Fos and the astrocyte-specific marker, S-100-protein β-subunit (S100), and (c) c-Fos and another astrocyte-specific marker, glial fibrillary acidic protein (GFAP). The results showed that most c-Fos-positive cells were NeuN-positive, S100-negative, and GFAP-negative, indicating that these c-Fos-expressing cells were primarily neurons (Fig. S2). However, we also identified a very small number of dual-positive cells for both c-Fos and S100, suggesting that astrocytes may express c-Fos under specific, rare conditions (Fig. S3).

We then conducted triple staining for c-Fos, S100, and the non-specific nuclear marker 4’,6-diamidino-2-phenylindole (DAPI) in four major cardiovascular brain regions: the central nucleus of the amygdala (CeA), the paraventricular nucleus of the hypothalamus (PVN), the dorsomedial hypothalamus (DMH), and the rostral ventral medulla (RVM). Additionally, we examined a non-cardiovascular brain region, represented by the deep ventral medulla (Figs. S4d, S6, S7). The expression of c-Fos in these regions was analyzed under four different conditions:

**Condition a**: Without arundic acid pre-treatment and without stress (Arundic acid −/Stress −, *n* = 2).

**Condition b**: Without arundic acid pre-treatment and with stress (Arundic acid −/Stress +, *n* = 3).

**Condition c**: With arundic acid pre-treatment and without stress (Arundic acid +/Stress −, *n* = 2).

**Condition d**: With arundic acid pre-treatment and with stress (Arundic acid +/Stress +, *n* = 2).

**Fig. 2:**
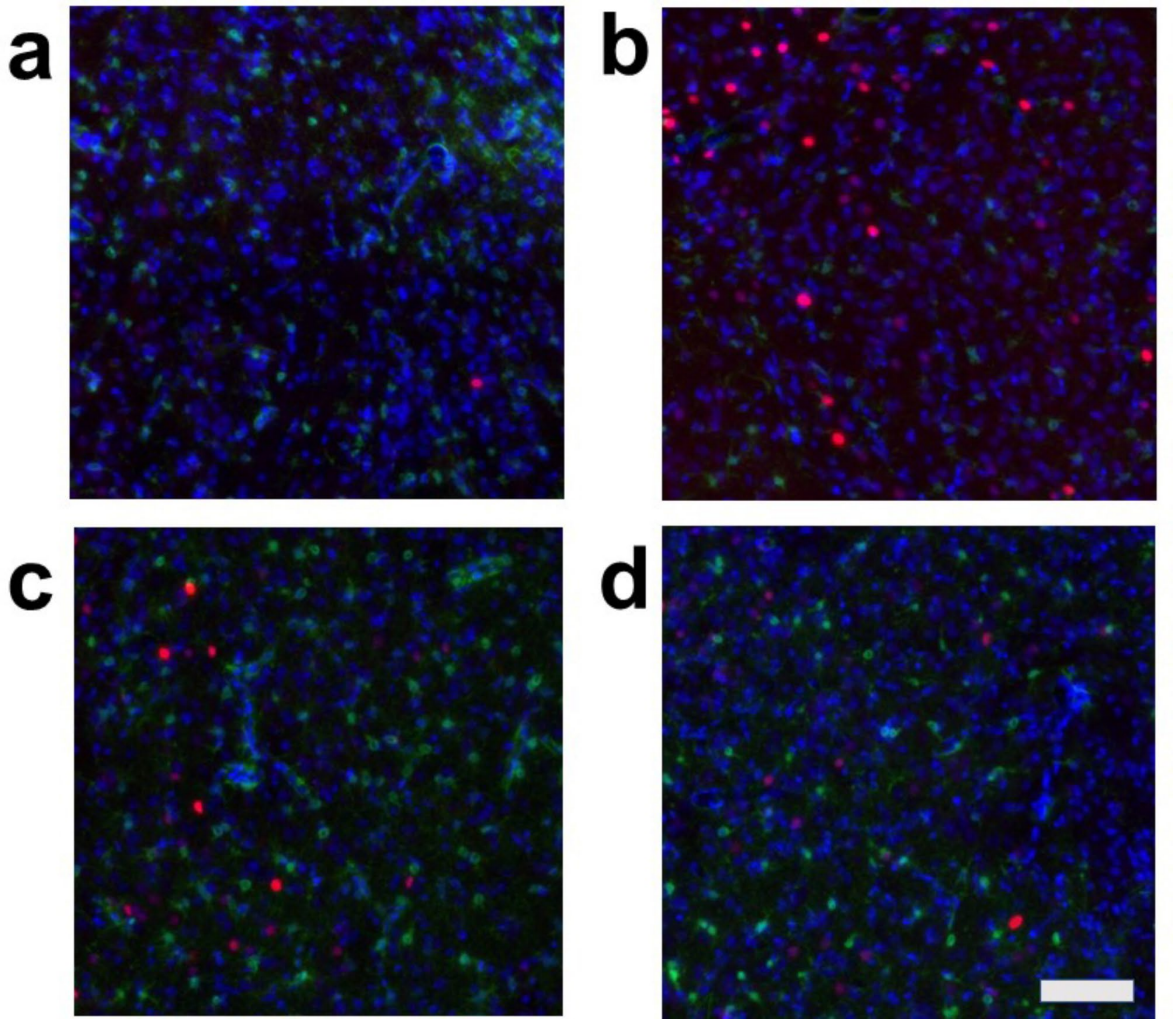
Double fluorescent immunostaining of c-Fos (red) and S100 (green) with DAPI (blue) staining in the central amygdala nucleus (CeA). (**a**) Condition **a** without arundic acid pre-treatment and without stress loading; (**b**) Condition **b**: without arundic acid pre-treatment and with stress loading; (**c**) Condition **c**: with arundic acid pre-treatment and without stress loading; (**d**) Condition **d**: with arundic acid pre-treatment and with stress loading. Under Condition **b**, a number of c-Fos positive cells were observed. The anatomical portion of the depicted brain region is shown in Fig. S3. Scale bar 100 μm.

**Fig. 3:**
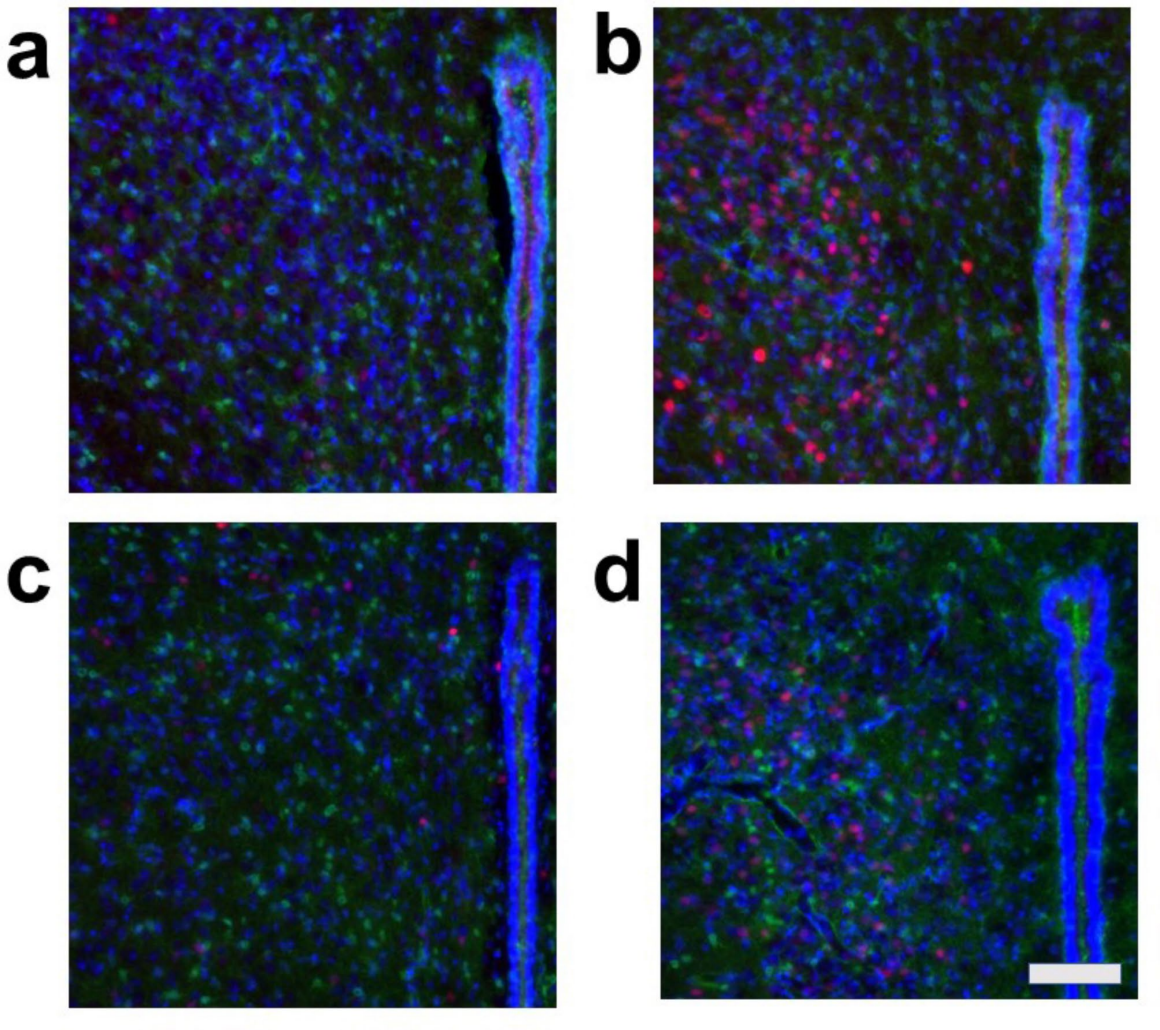
Double fluorescent immunostaining of c-Fos (red) and S100 (green) with DAPI (blue) staining in the paraventricular nucleus of the hypothalamus (PVN). (**a**) Condition **a** without arundic acid pre-treatment and without stress loading; (**b**) Condition **b**: without arundic acid pre-treatment and with stress loading; (**c**) Condition **c**: with arundic acid pre-treatment and without stress loading; (**d**) Condition **d**: with arundic acid pre-treatment and with stress loading. Under Condition **b**, a number of c-Fos positive cells were observed. The anatomical portion of the depicted brain region is shown in Fig. S3. Scale bar 100 μm.

**Fig. 4:**
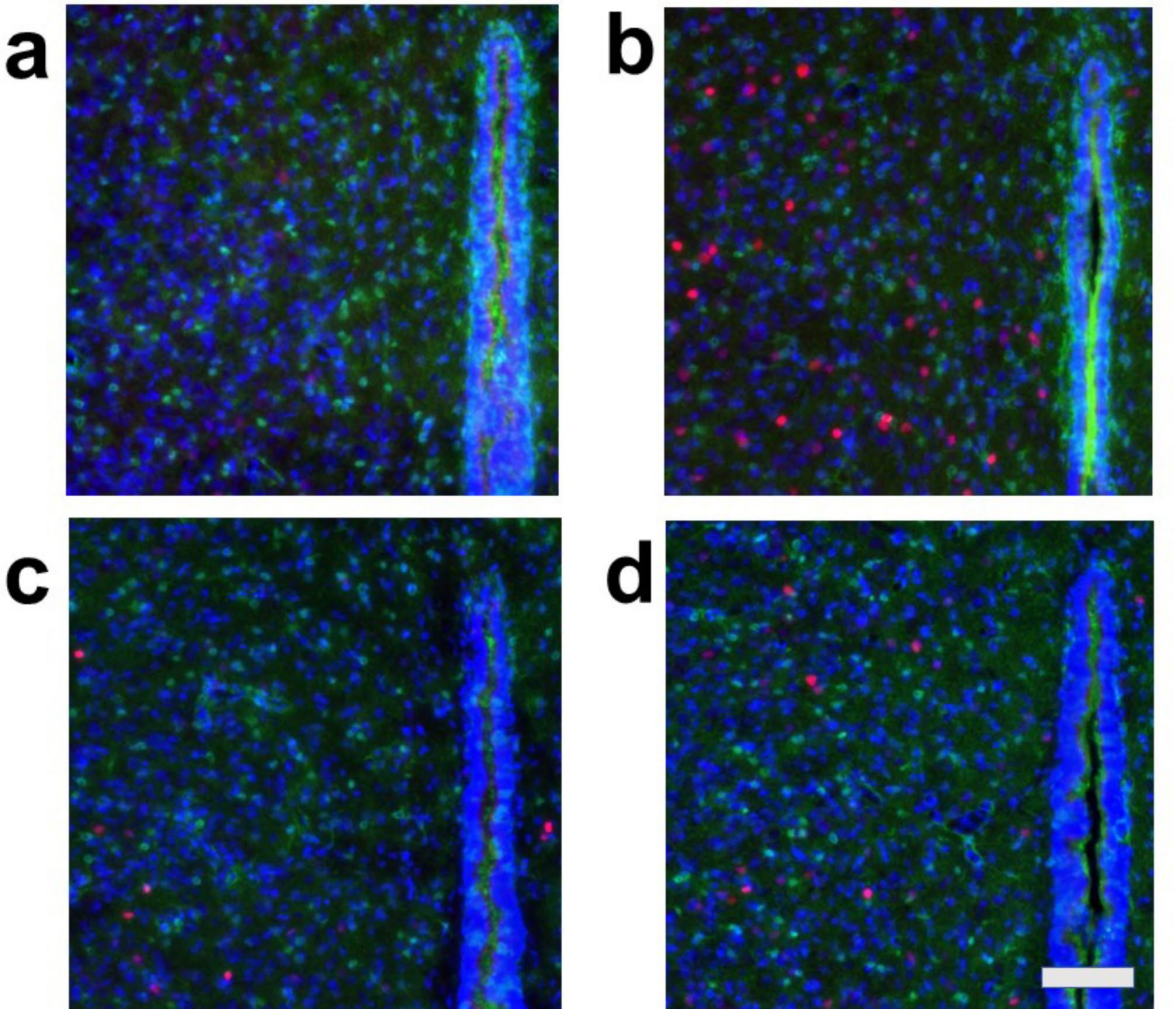
Double fluorescent immunostaining of c-Fos (red) and S100 (green) with DAPI (blue) staining in the dorsomedial hypothalamus (DMH). (**a**) Condition **a** without arundic acid pre-treatment and without stress loading; (**b**) Condition **b**: without arundic acid pre-treatment and with stress loading; (**c**) Condition **c**: with arundic acid pre-treatment and without stress loading; (**d**) Condition **d**: with arundic acid pre-treatment and with stress loading. Under Condition **b**, a number of c-Fos positive cells were observed. The anatomical portion of the depicted brain region is shown in Fig. S3. Scale bar 100 μm.

**Fig. 5:**
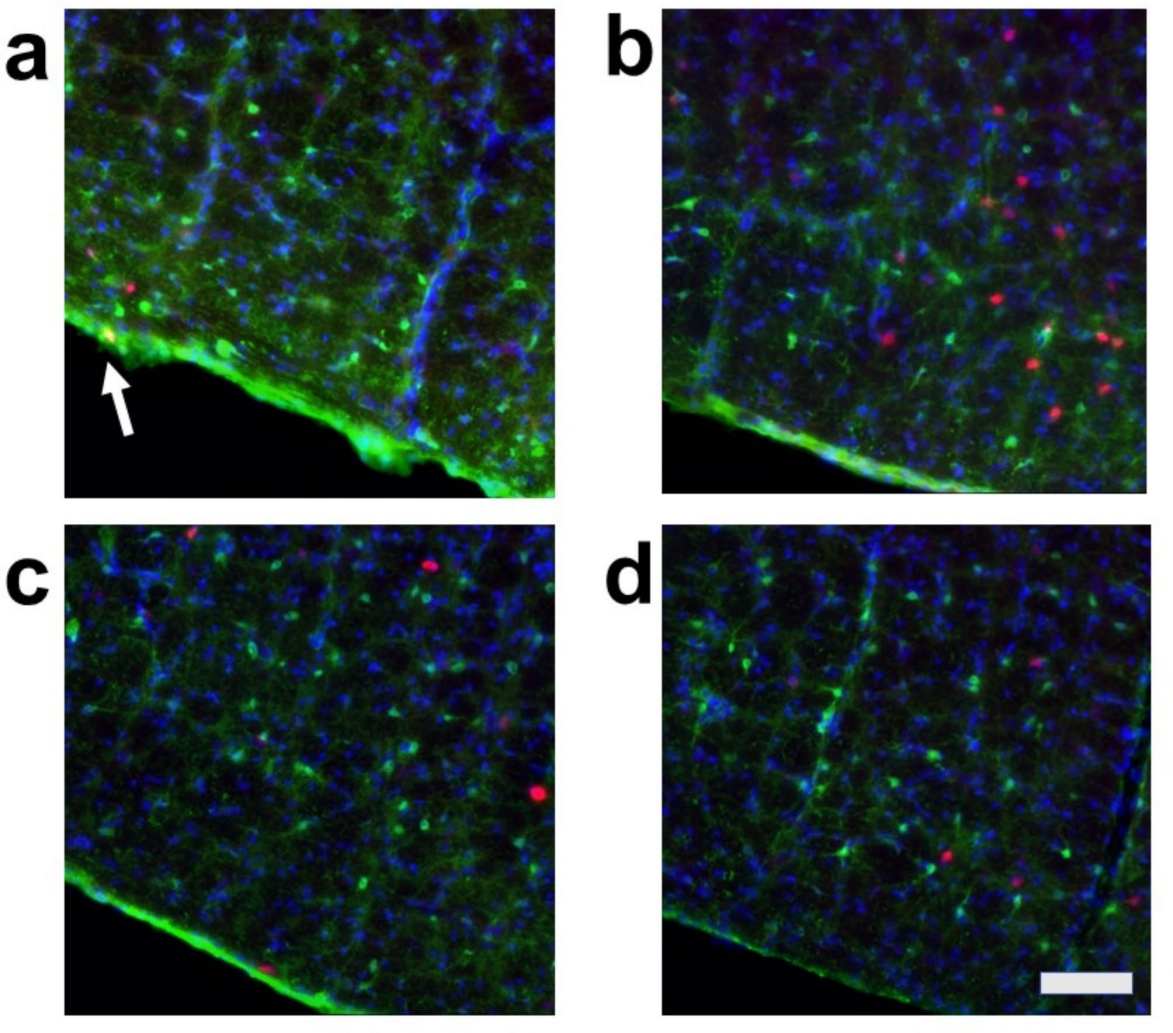
Double fluorescent immunostaining of c-Fos (red) and S100 (green) with DAPI (blue) staining in the rostral ventral medulla (RVM). (**a**) Condition **a** without arundic acid pre-treatment and without stress loading; (**b**) Condition **b**: without arundic acid pre-treatment and with stress loading; (**c**) Condition **c**: with arundic acid pre-treatment and without stress loading; (**d**) Condition **d**: with arundic acid pre-treatment and with stress loading. Under Condition **b**, a number of c-Fos positive cells were observed in the superficial ventral medullary region, i.e., in the cardiovascular region RVM. Double positivity for c-Fos and S100 (yellow in color) was rare, as exemplified by a cell under Condition **a** (arrow). Bottom oblique line, ventral medullary surface. The anatomical portion of the depicted brain region is shown in Fig. S3. Scale bar 100 μm.

Under all conditions, nearly all c-Fos-positive cells were S100-negative, indicating that these c-Fos-expressing cells were primarily neurons and not astrocytes (Figs. 2, 3, 4 and 5, Fig. S2). In the cardiovascular brain regions (CeA, PVN, DMH, and RVM), the density of c-Fos-positive cells was higher under Condition b compared to other conditions (Conditions a, c, and d) (Figs. 2, 3, 4 and 5, Fig. S5).

We also analyzed c-Fos expression in the non-cardiovascular brain region across all four conditions. In this region, specifically the rostral ventral medulla, air-jet stress did not induce c-Fos expression, although c-Fos was abundantly expressed in the RVM, a superficial ventral medullary cardiovascular region, as previously noted (Fig. S4b). Interestingly, under Condition c, the number of c-Fos-positive cells increased in the non-cardiovascular deep medullary region, suggesting that arundic acid alone (without stress) may have induced c-Fos expression in this area (Fig. S6c). All c-Fos-positive cells in the non-cardiovascular brain region were S100-negative, indicating that these cells were exclusively neurons (Fig. S6).

**Fig. 6:**
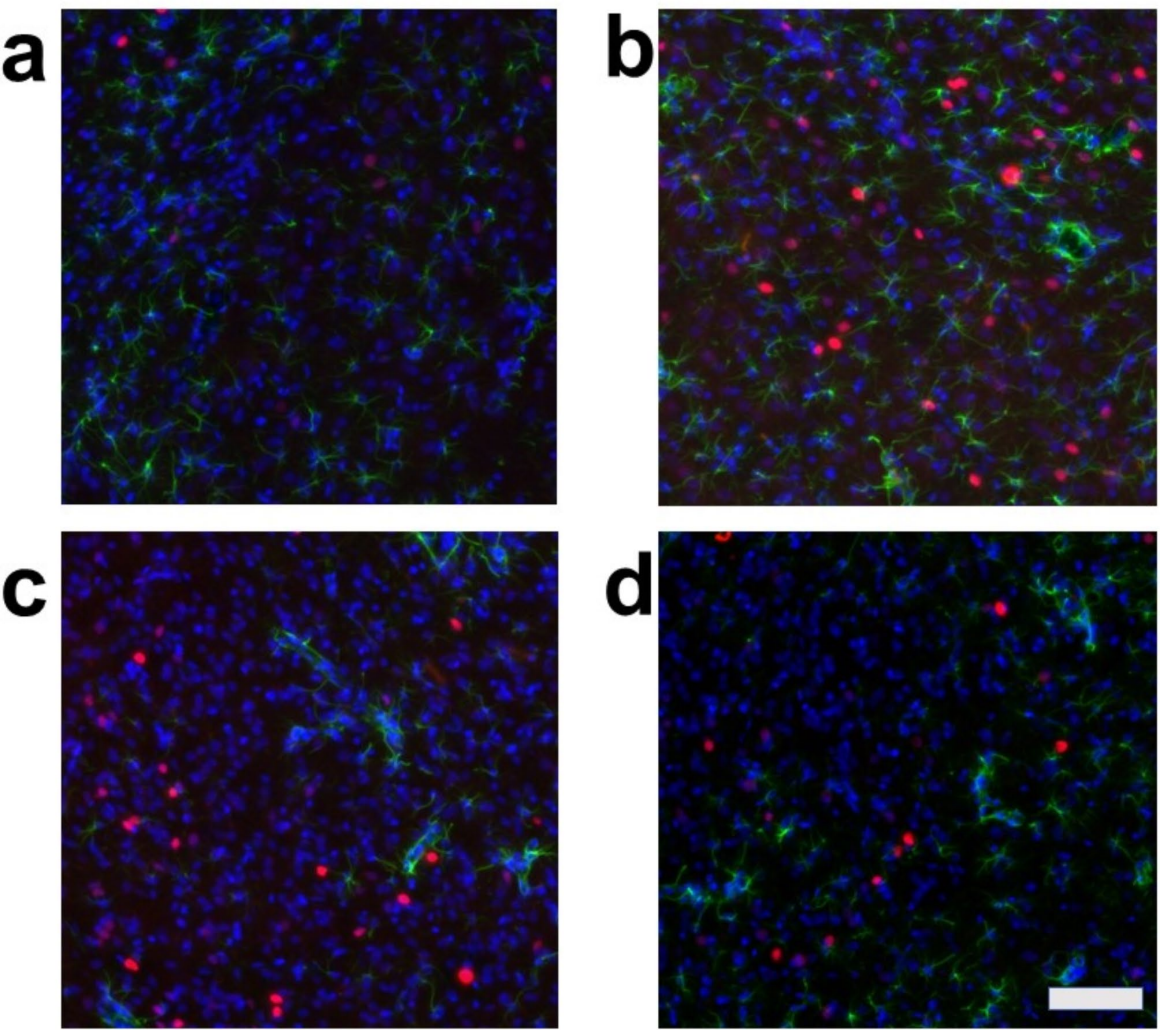
Double fluorescent immunostaining of c-Fos (red) and GFAP (green) with DAPI (blue) staining in the central amygdala nucleus (CeA). (**a**) Condition a without arundic acid pre-treatment and without stress loading; (**b**) Condition **b**: without arundic acid pre-treatment and with stress loading; (**c**) Condition **c**: with arundic acid pre-treatment and without stress loading; (**d**) Condition **d**: with arundic acid pre-treatment and with stress loading. Under Conditions **b** and **c**, a number of c-Fos positive cells were observed. The anatomical portion of the depicted brain region is shown in Fig. S3. Scale bar 100 μm.

**Fig. 7:**
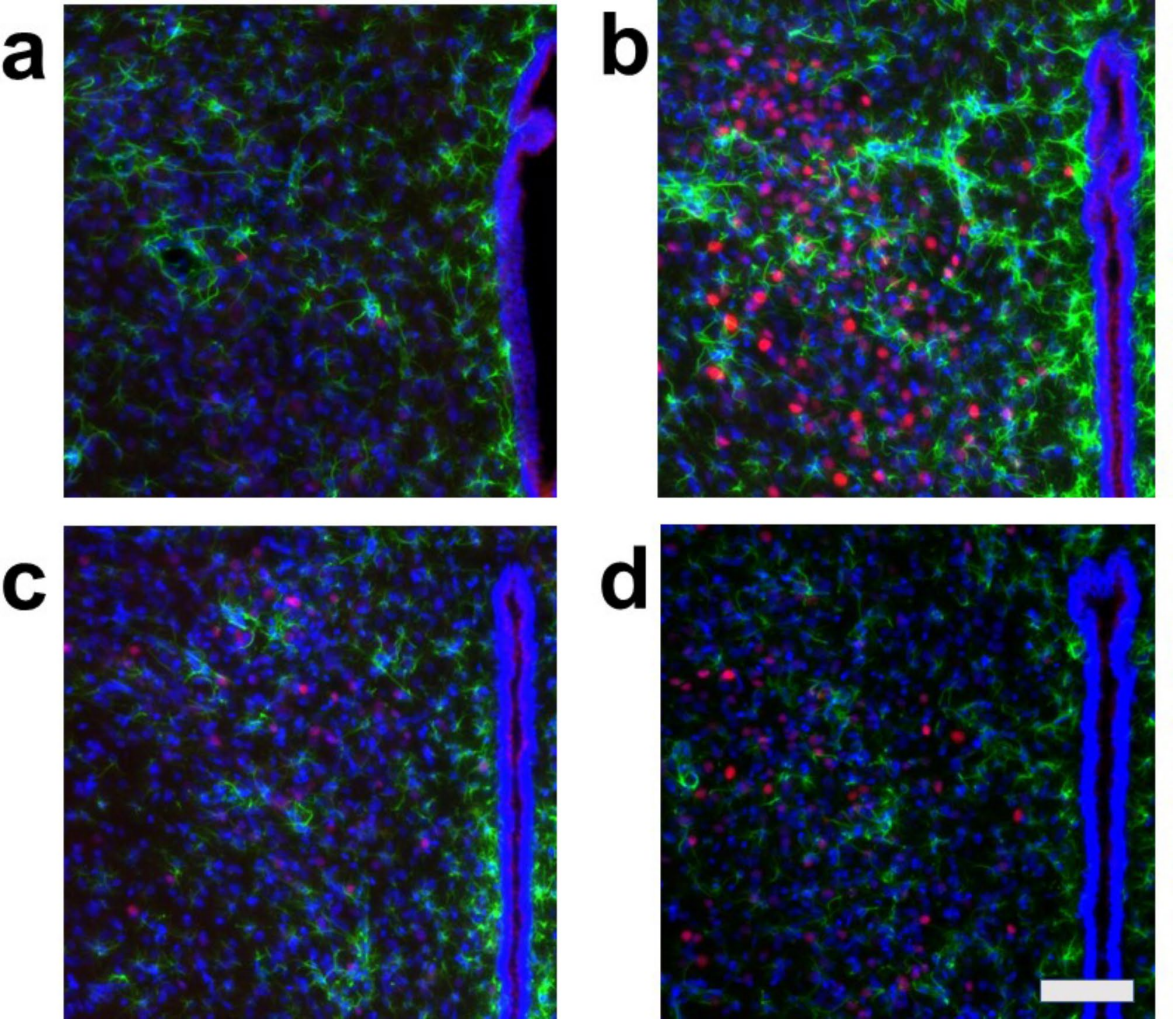
Double fluorescent immunostaining of c-Fos (red) and GFAP (green) with DAPI (blue) staining in the paraventricular nucleus of the hypothalamus (PVN). (**a**) Condition **a** without arundic acid pre-treatment and without stress loading; (**b**) Condition **b**: without arundic acid pre-treatment and with stress loading; (**c**) Condition **c**: with arundic acid pre-treatment and without stress loading; (**d**) Condition **d**: with arundic acid pre-treatment and with stress loading. Under Condition **b**, a number of c-Fos positive cells were observed. The anatomical portion of the depicted brain region is shown in Fig. S3. Scale bar 100 μm.

**Fig. 8:**
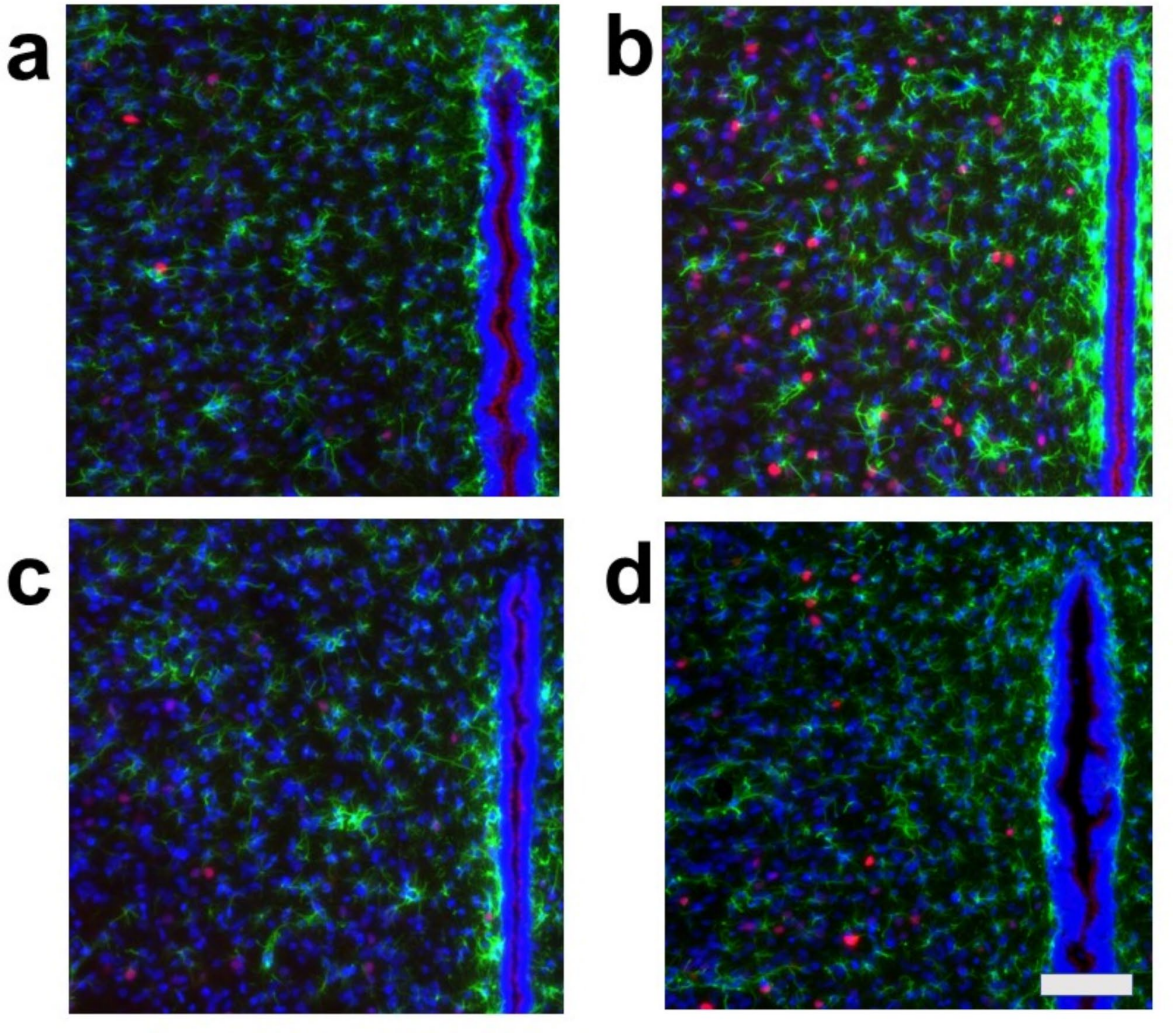
Double fluorescent immunostaining of c-Fos (red) and GFAP (green) with DAPI (blue) staining in the dorsomedial hypothalamus (DMH). (**a**) Condition **a** without arundic acid pre-treatment and without stress loading; (**b**) Condition **b**: without arundic acid pre-treatment and with stress loading; (**c**) Condition **c**: with arundic acid pre-treatment and without stress loading; (**d**) Condition **d**: with arundic acid pre-treatment and with stress loading. Under Condition **b**, a number of c-Fos positive cells were observed. The anatomical portion of the depicted brain region is shown in Fig. S3. Scale bar; 100 μm.

**Fig. 9:**
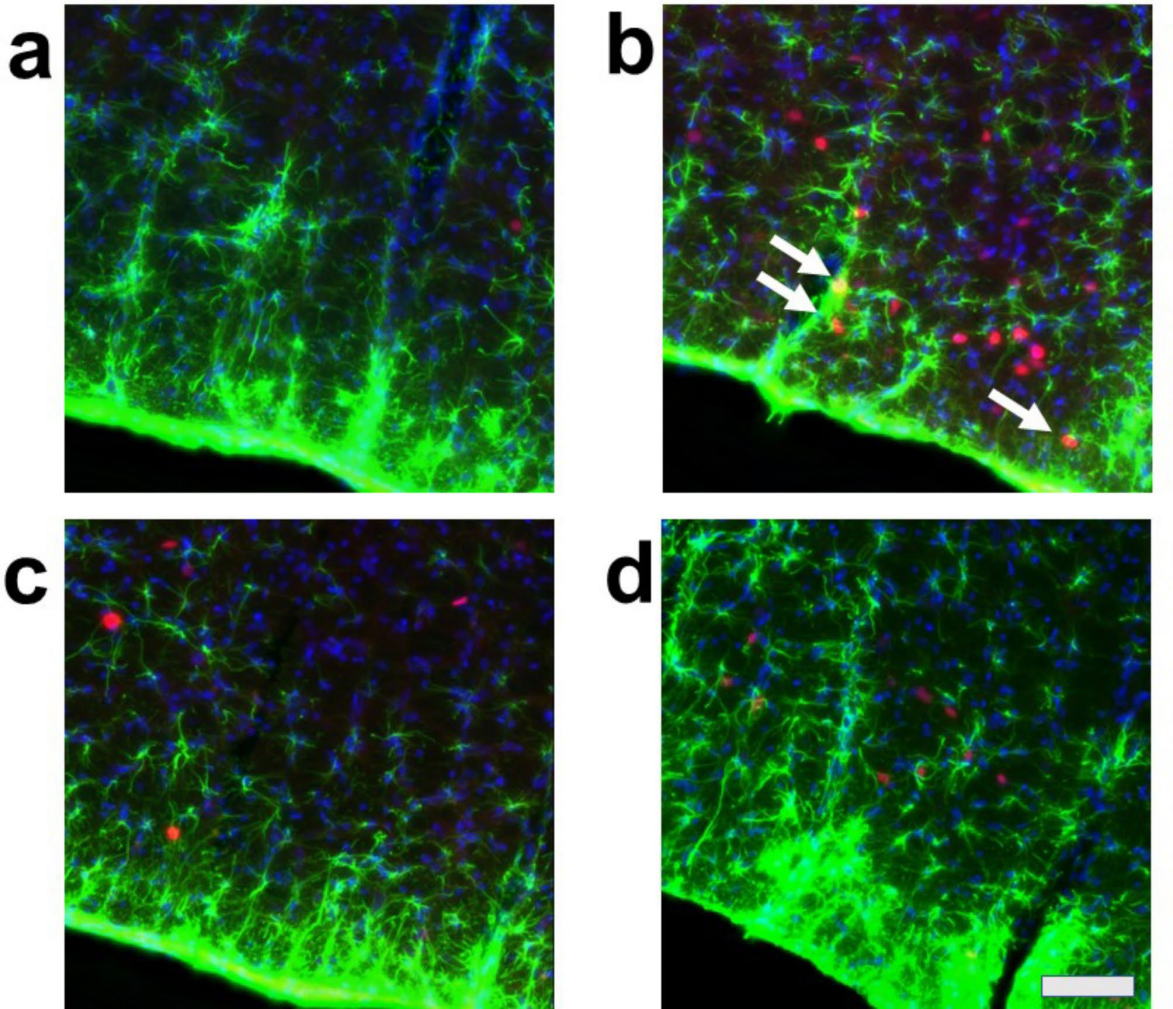
Double fluorescent immunostaining of c-Fos (red) and GFAP (green) with DAPI (blue) staining in the rostral ventral medulla (RVM). (**a**) Condition **a** without arundic acid pre-treatment and without stress loading; (**b**) Condition **b**: without arundic acid pre-treatment and with stress loading; (**c**) Condition **c**: with arundic acid pre-treatment and without stress loading; (**d**) Condition **d**: with arundic acid pre-treatment and with stress loading. GFAP positive fibers were densely distributed just beneath the ventral medullary surface and constituted the glia limitans (marginal glial layer). Under Condition **b**, a number of c-Fos positive cells were observed in the superficial ventral medullary region, i.e., in the cardiovascular region RVM, Although the cells with arrows appeared yellow or orange, they were not double-positive for c-Fos and GFAP; their c-Fos-positive nuclei and GFAP-positive structures were not precisely aligned on the same z-axis planes. Bottom oblique line, ventral medullary surface. The anatomical portion of the depicted brain region is shown in Fig. S3. Scale bar; 100 μm.

Because we rarely observed c-Fos and S100 double-positive cells, we further examined whether astrocytes express c-Fos by using GFAP, another astrocyte marker. We performed triple staining for c-Fos, GFAP, and DAPI in cardiovascular brain regions, including the CeA, PVN, DMH, and RVM, as well as in the same non-cardiovascular brain region, under the same four conditions. This protocol mirrored the one used for triple staining with c-Fos, S100, and DAPI. In all cardiovascular brain regions analyzed (CeA, PVN, DMH, and RVM), the density of c-Fos-positive cells was higher under Condition **b** (without arundic acid pre-treatment and with stress) compared to other conditions (Conditions **a**, **c**, and **d**) (Figs. 6, 7, 8 and 9, Fig. S4). Almost all c-Fos-positive cells were GFAP-negative across all conditions, indicating that these c-Fos-expressing cells were predominantly neurons (Figs. 6, 7, 8 and 9). We also analyzed c-Fos expression in cells simultaneously stained with GFAP and DAPI in the non-cardiovascular brain region under the four different conditions. Air-jet stress did not induce c-Fos expression in the non-cardiovascular medullary regions, although c-Fos was abundantly expressed in the cardiovascular RVM region (Fig. S7b). Interestingly, under Condition **c** (with arundic acid pre-treatment and without stress), the number of c-Fos-positive cells increased in the non-cardiovascular deep medullary region, suggesting that arundic acid alone induced c-Fos expression in this area (Fig. S7c). Similar to the cardiovascular regions, all c-Fos-positive cells in the non-cardiovascular brain region were GFAP-negative, indicating that these cells were exclusively neurons (Fig. S7). In this immunohistological analysis, we examined brain tissue from each region (CeA, PVN, DMH, RVM, and non-cardiovascular deep medullary areas) under all conditions using samples from at least two rats per condition, and consistent results were obtained across all groups.

**Fig. 10:**
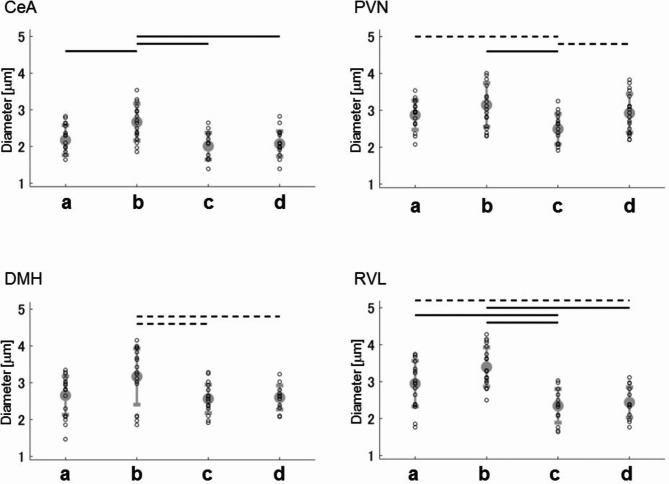
Diameters of the first-order processes of astrocytes in the cardiovascular brain areas CeA, PVN, DMH and RVM under each drug condition. The diameter of astrocytic first-order processes under Condition **b** (without arundic acid, with stress) tended to be consistently larger than under other conditions, suggesting that air-jet stress activated astrocytes in the cardiovascular brain areas. This activation was suppressed by pre-treatment with arundic acid. (**a**) Condition **a** without arundic acid pre-treatment and without stress loading; (**b**) Condition **b**: without arundic acid pre-treatment and with stress loading; (**c**) Condition **c**: with arundic acid pre-treatment and without stress loading; (**d**) Condition **d**: with arundic acid pre-treatment and with stress loading. Horizontal dotted lines indicate the pairs that are statistically different, Bonferroni corrected *p* < 0.05. Horizontal dotted lines indicate statistically significant differences (Bonferroni corrected, *p* < 0.05), while horizontal solid lines indicate more robust statistical significance (Bonferroni corrected, *p* < 0.01).

To assess the degree of astrocytic activation, we quantitatively analyzed astrocyte morphology, as c-Fos immunohistology alone was not sufficient for this purpose. Specifically, we examined the thickness of astrocytic first-order processes, as this measure is known to reflect the level of cell activation^23,24^. In the cardiovascular brain regions (CeA, PVN, DMH, and RVM), the diameters of astrocytic first-order processes under Condition **b** (without arundic acid, with stress) were consistently larger than those observed under other conditions. This suggests that air-jet stress activated astrocytes in these cardiovascular regions, and that pre-treatment with arundic acid effectively suppressed this astrocytic activation (Fig. 10, Fig. S8).

### Effect of arundic acid on blood pressure

**Fig. 11:**
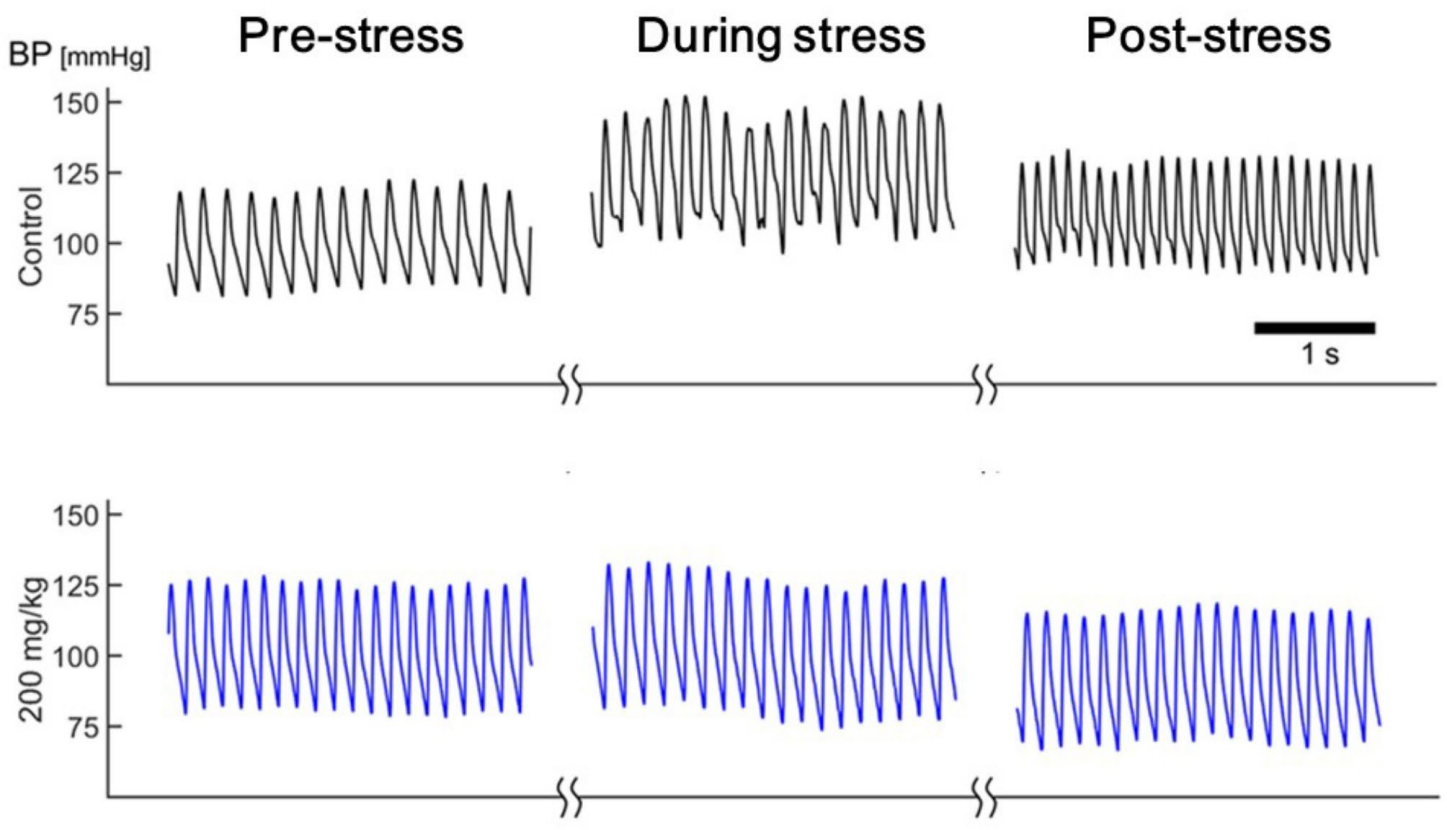
Representative blood pressure recordings during pre-stress, stress loading and post-stress periods in a rat under each drug condition. In the pre-stress resting state, blood pressure did not differ across drug conditions. Under the control condition (without arundic acid pre-treatment), the rat exhibited significant blood pressure elevation with fluctuations during stress, which persisted throughout the post-stress observation period. After arundic acid pre-treatment, blood pressure elevation during stress loading was reduced, and the blood pressure tended to be lower once the stress loading ended. The “during stress” panel represents 1 min after the onset of stress loading; the “post-stress” panel represents 8 min after the end of stress loading. Time bar: 1 s.

**Fig. 12:**
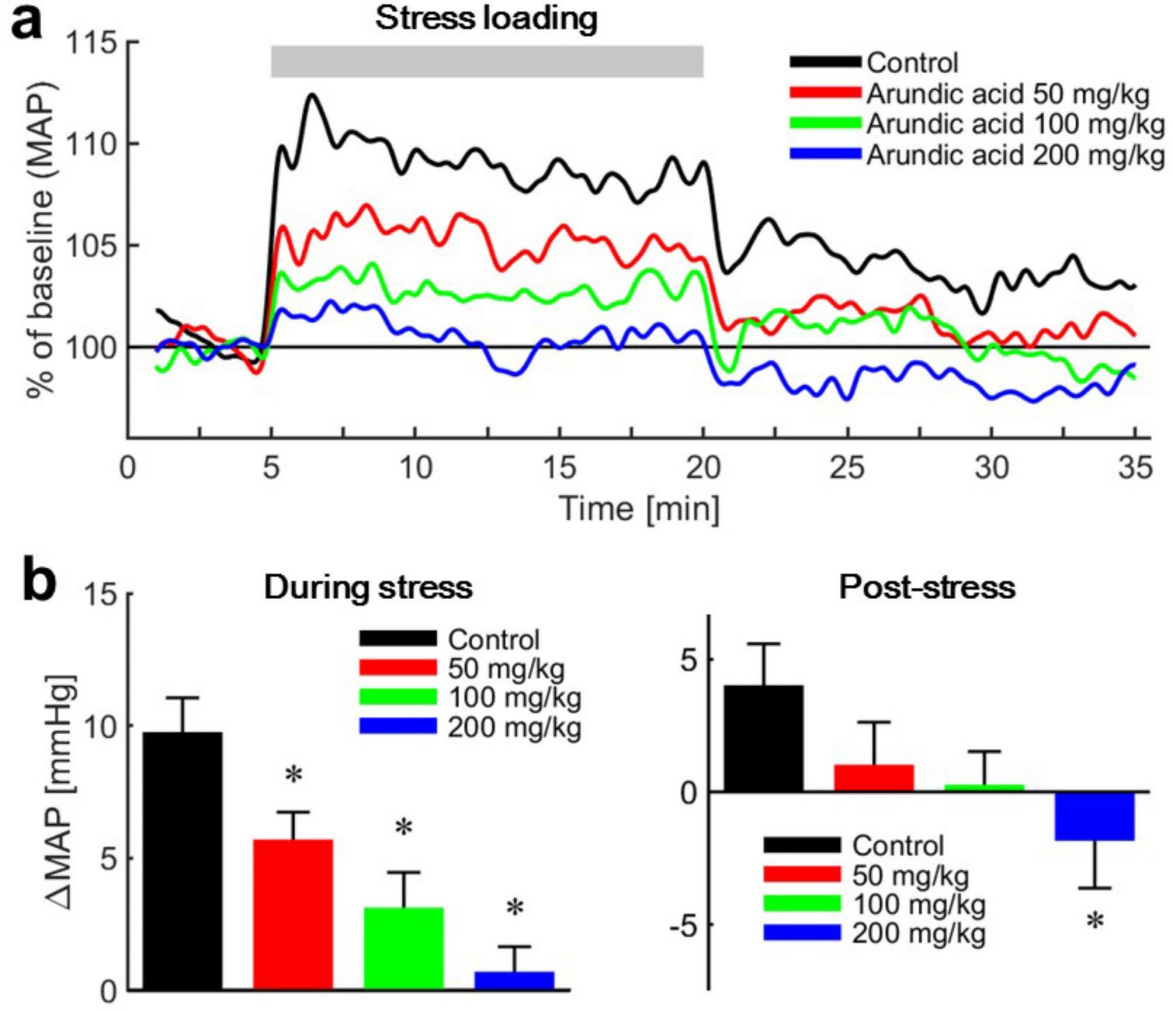
Changes in mean arterial blood pressure (MAP). (**a**) Time course of MAP before, during, and after stress loading. MAP values obtained from all tested rats were averaged and normalized to baseline (MAP recorded for 2.5 min before stress loading) and set as 100%. Stress-induced blood pressure elevation during and after stress loading was suppressed by prior administration of arundic acid in a dose-dependent manner. (**b**) Average change in MAP (ΔMAP) during air-jet stress (left) and post-stress (right) periods compared to the pre-stress resting state. ΔMAP during stress was significantly diminished by arundic acid pre-treatment in a dose-dependent manner. Although ΔMAP during the post-stress period remained positive under the control condition, it decreased and became negative with arundic acid pre-treatment in a dose-dependent manner. Data are presented as means ± SE, *n* = 13 per group. *Bonferroni corrected, *p* < 0.05 vs. control group.

The mean arterial blood pressure (MAP) did not change significantly before and after the administration of arundic acid (at doses of 50, 100, or 200 mg/kg) prior to stress loading (Table 1). Air-jet stress caused an immediate rise in MAP under both control and arundic acid-treated conditions (Figs. 11 and 12). However, the increase in MAP, measured as the difference between pre-stress and stress-loading levels (ΔMAP), was significantly smaller in all arundic acid-treated groups compared to the control group without arundic acid pre-treatment (Fig. 12). The reduction in stress-induced blood pressure elevation was dose-dependent.

**Table 1:** Effects of arundic acid on resting blood pressure and heart rate.

	Doses of arundic acid (mg/kg)			
	0	50	100	200
MAP (mmHg)	113 ± 4	114 ± 4	116 ± 3	115 ± 3
HR (beat/min)	403 ± 21	411 ± 19	395 ± 13	386 ± 19

Baseline values were calculated as the 2.5-min average of mean arterial blood pressure (MAP) and heart rate (HR) before air-jet stress loading. No significant differences were observed in either MAP or HR across the different arundic acid doses. Data are presented as means ± SE (*n* = 13).

When the air-jet stress was stopped, MAP decreased under all conditions. In the control group, MAP rebounded 1–2 min after the stress ended, followed by a gradual decline, but it remained above the baseline level throughout the 15-minute post-stress observation period (Fig. 11a). In the medium- and high-dose arundic acid groups (100 and 200 mg/kg), MAP dropped immediately below baseline when the stress ended. After this initial post-stress drop, MAP in the high-dose (200 mg/kg) group remained below baseline for the rest of the observation period (Fig. 12).

### Effect of arundic acid on HR

**Fig. 13:**
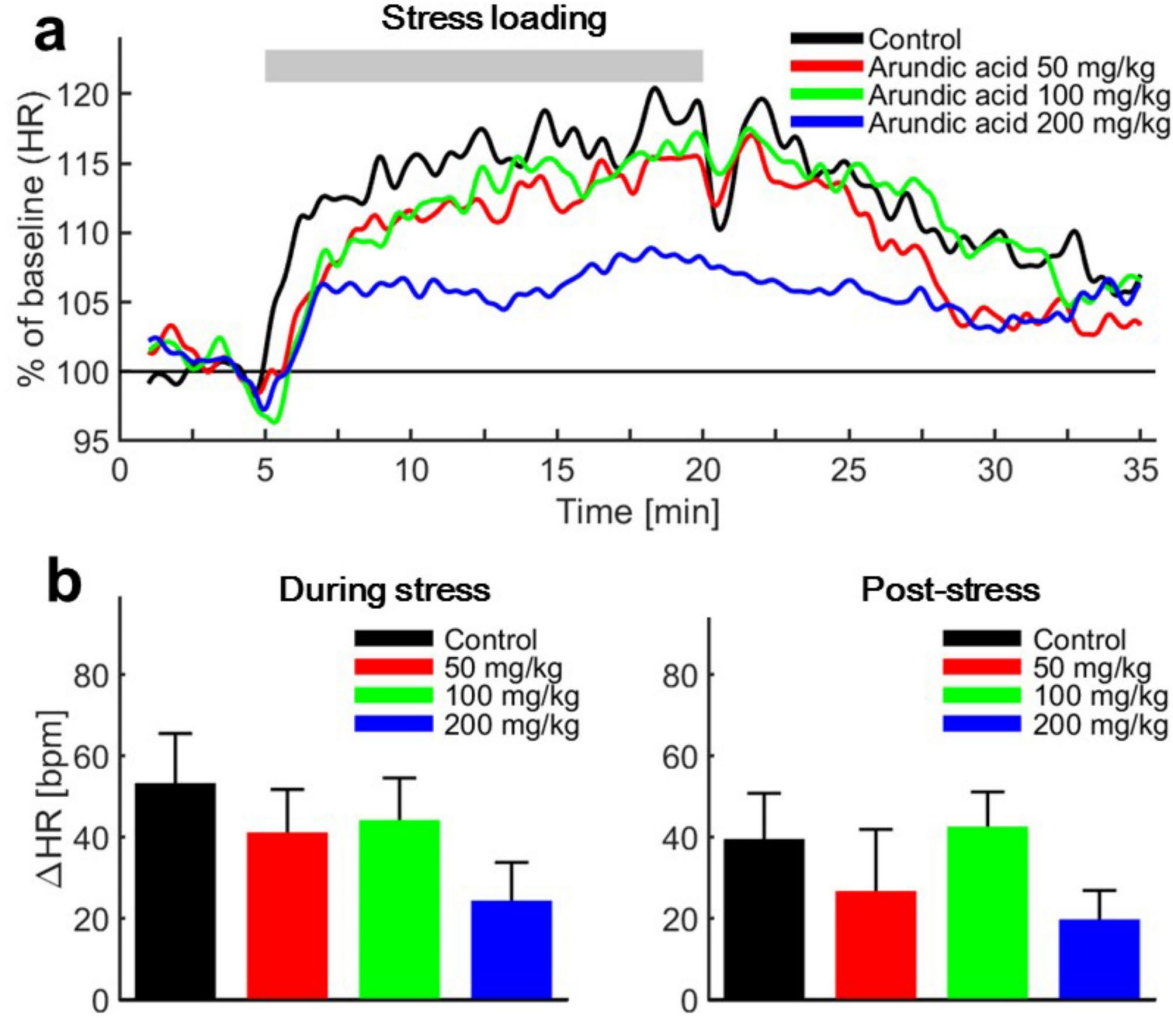
Changes in heart rate (HR). (**a**) Time course of HR before, during, and after stress loading. HR values from all tested rats were averaged and normalized to baseline (HR recorded for 2.5 min before stress loading) and set as 100%. Stress-induced HR increase was suppressed by prior administration of a representative high dose of arundic acid (200 mg/kg), although the effect of arundic acid on HR responses to stress was not dose-dependent. (**b**) Average change in HR (ΔHR) during air-jet stress (left) and post-stress (right) periods compared to the pre-stress resting state. There was no significant difference compared to the control in any period. Data are presented as means ± SE, *n* = 13 per group.

Similarly, HR showed no significant differences before and after the administration of arundic acid (at doses of 50, 100, or 200 mg/kg) prior to stress loading (Table 1). Air-jet stress caused an immediate increase in HR under both control and arundic acid-treated conditions (Fig. 13a). The degree of HR increase, measured as the difference between pre-stress and stress-loading levels (ΔHR), did not vary significantly among the groups treated with different doses of arundic acid.

When air-jet stress was terminated, HR immediately decreased for a short period in all groups, except for the high-dose (200 mg/kg) arundic acid group, which showed a delayed response. After 1–2 min, HR began to increase again before gradually declining. However, HR remained above baseline in all groups throughout the post-stress observation period (Fig. 13). Although HR in the high-dose (200 mg/kg) group tended to be lower than in the other groups during and after stress loading, these differences were not statistically significant (Fig. 13b).

## Discussion

In this study, we first confirmed that arundic acid specifically inhibits astrocytes in a dose-dependent manner and does not affect neurons. Immunohistochemical analyses revealed that air-jet stress induced abundant c-Fos expression exclusively in neurons within the cardiovascular brain regions of the amygdala, hypothalamus, and medulla oblongata. This stress-induced neuronal activation was suppressed by pre-treatment with arundic acid. Additionally, by measuring the thickness of astrocytic processes, we confirmed that astrocytes in these cardiovascular brain regions were activated by stress, and this activation was blocked by the pre-administration of arundic acid. In unanesthetized, unrestrained rats, air-jet stress elevated both blood pressure and HR. These increases persisted for a considerable time after the stress loading was terminated. Pre-treatment with arundic acid suppressed the stress-induced elevation in blood pressure both during and after stress loading. Although arundic acid did not significantly suppress HR increases during and after stress, HR in the high-dose (200 mg/kg) arundic acid group tended to be lower than in other dose groups. However, arundic acid pre-treatment had no effect on blood pressure or HR under resting conditions before stress loading. These findings suggest that activated astrocytes play a key role in stress-induced blood pressure elevation and its persistence after stress. In contrast, astrocytes that are not activated do not appear to be critical for the maintenance of blood pressure or HR under resting conditions.

Arundic acid inhibits the function of astrocytes by blocking the synthesis of S100 and suppressing the cytokine cycle^17,18,20,22^. It has also been reported to increase the expression of glutamate transporters in astrocytes^19,25^. In this study, we confirmed through ratiometric calcium imaging in cultured astrocytes and neurons that arundic acid’s inhibitory action is specific to astrocytes (Fig. 1). This was further validated using calcium imaging in acute brain slices from young adult rats (Fig. S1). We also assessed cellular activation in cardiovascular brain regions in response to air-jet stress using c-Fos immunohistology. While the interpretation of these results may be somewhat complex, they can be logically explained as follows: (1) Without arundic acid pre-treatment, air-jet stress led to abundant c-Fos expression in neurons in the cardiovascular brain regions of the amygdala, hypothalamus, and medulla oblongata, indicating that air-jet stress activates neurons in these regions. (2) With arundic acid pre-treatment, this stress-induced c-Fos expression in neurons was attenuated, suggesting that arundic acid inhibits neuronal activation in these regions. (3) In the earlier part of the study, we showed that arundic acid specifically blocks external stimulation-induced astrocytic activation without affecting neuronal excitation. (4) Collectively, these findings suggest that astrocytes play a crucial role in mediating stress-induced neuronal activation.

Although astrocytes are capable of expressing c-Fos^26,27^, in our immunohistochemical analyses, air-jet stress rarely induced c-Fos expression in astrocytes within the cardiovascular brain regions. Nearly all c-Fos-positive cells were identified as neurons (Figs. S1, S2, Figs. 2, 3, 4, 5, 6, 7, 8 and 9). It is important to note that the conditions required for astrocytes to express c-Fos differ from those for neurons, whose expression changes are associated with depolarization. Astrocytes do not express c-Fos in response to depolarizing stimuli but rather in contexts involving proliferation, differentiation, growth, inflammation, repair, damage, and plasticity^27^.

In the cardiovascular regions, the CeA, PVN, DMH and RVM, neurons abundantly expressed c-Fos under the condition without arundic acid pre-treatment and with stress loading (Condition **b**) (panels **b** of Figs. 2, 3, 4, 5, 6, 7, 8 and 9). Such augmented c-Fos expression was suppressed by arundic acid pre-treatment (Condition **d**) (panels **d** of Figs. 2, 3, 4, 5, 6, 7, 8 and 9). Thus, the histologically observed suppression of air-jet stress-induced activation of neurons by pre-treatment with arundic acid, i.e., decreases in c-Fos positive cells under Condition **d**, could be attributed to the diminished excitatory signals from astrocytes to neurons in the local astrocyte-neuron network in each cardiovascular region. This notion is in agreement with the reports that psychological stress activates not only neurons but astrocytes and the activated astrocytes modulate the function of neurons within the local astrocyte-neuron network^28–30^.

In contrast to the cardiovascular regions, stress-evoked c-Fos expression was not observed in the non-cardiovascular regions, at least in the deep ventral medullary area analyzed (Figs. S6b, S7b), suggesting that the stress stimulation used in this study did not affect neurons in these regions. However, we did observe c-Fos expression in the non-cardiovascular deep ventral medullary region under conditions with arundic acid, even without stress loading (Figs. S6c, S7c). The mechanism underlying this c-Fos expression is unclear, but it may be related to the neuron-stimulating effects of arundic acid (Fig. 1c,d). While speculative, it is possible that neurons in these regions are sensitive to arundic acid. Nevertheless, these findings do not affect the interpretation of the main results in this study, which focus on the suppression of stress-induced neuronal activation in the cardiovascular brain regions mediated by arundic acid.

When a subject is exposed to psychological stress, cortical and limbic neuronal networks, including those of the amygdala, are instantly activated. This information is integrated mainly in the CeA and subsequently sent to the more caudal brain regions that govern the cardiovascular regulation^31–35^. Among the regions involved in cardiovascular regulation, the hypothalamus has been recognized as a key forebrain region, with the PVN and DMH being of particular importance^36–38^. The PVN coordinates the autonomic nervous and hypothalamo-neurohypophysial neuroendocrine systems. When the PVN is activated, its neurons increase the production of vasopressin which acts not only as a circulatory hormone but also as a neurotransmitter that directly excites sympathetic preganglionic neurons, resulting in acute blood pressure elevation^37,38^. It has also been reported that when astrocytes in the PVN are activated they release ATP and elevate blood pressure^39^. The DMH provides neuronal projections to the PVN^40^ and RVM^36,41–43^, mediating the stress-related information for blood pressure elevation and HR increase. The RVM has been recognized as an essential brain region for sympathetic cardiovascular regulation^32,34,41,44,45^, where neurons richly express angiotensin II type 1 receptor (AT1R)^44,46^.

Recently, astrocytes have been increasingly recognized to play a role in the central control of blood pressure and HR^47–50^. In various brain regions, neurons activated by acutely loaded stress secrete various neurotransmitters, e.g., glutamate and ATP, in the paracrine fashion. These neurotransmitters not only excite postsynaptic neurons but also activate astrocytes surrounding synapses. Then, astrocytes respond by increasing the intracellular calcium concentration, and persistently augment the excitability of surrounding neurons through the release of gliotransmitters^8,16,47,48,51–55^ and/or through the transient expression of metabotropic glutamate receptor 5 in astrocytes^14^. For example, the release of glutamate, D-serine, L-lactate or ATP from astrocytes promotes excitatory communication between neurons and astrocytes^8,11,16,47,48,51^. These phenomena occur within a few seconds triggered by neuronal activation but persist longer owing to sustained activation of astrocytes^56,57^. Thus, we presume that the psychological stress employed in the present study activated sympathoexcitatory neurons and astrocytes in the cardiovascular regions, particularly the amygdala, hypothalamus and medulla, resulting in sustained blood pressure elevation and HR increase. The presumption of astrocytic involvement is supported by decreases in c-Fos positive cells in the CeA, PVN, DMH and RVM regions, most of which were presumably neurons, following pre-treatment with arundic acid, which specifically inhibits astrocytic activation. The stress-induced sustained activation of sympathoexcitatory neurons and astrocytes may underlie the pathogenesis of hypertension. Likewise, the pre-treatment with high-dose arundic acid tended to suppress the stress-evoked increase in HR during and after stress loading, however, the degree of this suppression was not statistically significant. The less apparent suppressive effect of arundic acid on HR than on blood pressure could be explained at least partly by different control mechanisms of blood pressure and HR in the brain^58^. The finding of the present study that arundic acid per se did not affect resting-state blood pressure or HR before stress loading suggests that astrocytes do not play a critical role in the maintenance of sympathetic activity under the resting condition in healthy non-hypertensive animals (Table 1). However, in spontaneously hypertensive animals, astrocytes in the cardiovascular brain regions may be persistently activated even without stress loading and may be responsible for sustained sympathoexcitation. This explanation is supported by the observation that hypertension in stroke-prone spontaneously hypertensive rats is partially ameliorated by chronic (more than 8 weeks long) administration of arundic acid^59^, by histological observation of astrogliosis in spontaneously hypertensive rats^49^ and by the observations that the functional properties of astrocytes isolated from spontaneously hypertensive rats, especially the responsiveness of these astrocytes to angiotensin II, are different from those of normotensive Wistar rats^59–62^. Notably, chronic overnutrition (or astrocytic IKKβ/NF-κB upregulation) causes sustained shortening of astrocytic processes in the hypothalamus, which results in blood pressure elevation and body weight and fat gain^63^. These findings suggest that chronically-activated hypothalamic astrocytes play a role in the pathogenesis of hypertension in patients with metabolic syndrome.

In the present study, we could not identify the direct causative factors, i.e., gliotransmitters, from astrocytes that induce neuronal alteration in the stress-loaded animals. Although this issue is a topic necessary to be studied in the future, the renin-angiotensin system (RAS) may be critically involved as discussed below. The peripheral RAS is a well-recognized key player in cardiovascular regulation. Although angiotensin II in the peripheral blood does not penetrate the blood-brain barrier under normal conditions^64^, the brain RAS has been increasingly investigated for its involvement in central cardiovascular regulation. Angiotensinogen is produced in the brain almost exclusively by astrocytes and is catalyzed to angiotensin I by the prorenin receptor or renin and further to angiotensin II by brain angiotensin converting enzyme^64–68^. The level of brain angiotensin II increases in response to stress, and angiotensin II acts at AT1R which is abundantly expressed in the cardiovascular brain regions^69^. AT1R-expressing cells in the brain are predominantly neurons, but angiotensin II acts also at AT1R on astrocytes, resulting in blood pressure elevation^70,71^ and a HR increase^72^. Angiotensin II also stimulates vasopressin secretion in the hypothalamus^37,73^. Thus, angiotensin II acts not only in a paracrine (from astrocyte to neuron) but also in an autocrine (from astrocyte to astrocyte) manner. The findings in the present study, along with the previous data outlined above, strongly suggest that psychological stress activates sympathetic neurons followed by longer-lasting activation of surrounding astrocytes, possibly facilitating angiotensinogen secretion and sustained blood pressure elevation. This notion is also supported by previous reports that enforced enhancement of RAS in astrocytes elevates blood pressure^71,74^ and that astrocyte-specific ablation of angiotensinogen lowers blood pressure in mice^66^ and rats^67^. Suppression of the overexcited local RAS in the brain could be a strategy for drug development for hypertension, targeting astrocytes in the cardiovascular brain regions, such as a blood-brain barrier penetrating AT1R blocker^75^ or a blocker of astrocytic activation^59^.

In the present study, we focused our attention on neurons and astrocytes, but microglia are also involved in central information processing under physiological and pathological conditions^76–78^. Therefore, microglia may also be involved in the stress-induced sympathoexcitatory responses by interacting with neurons and astrocytes in the cardiovascular brain regions^45,48,79–83^. The pathophysiological mechanism of neurogenic hypertension, especially in stressed individuals, may be explained by positive feedback among neurons, astrocytes and microglia; an area of limited understanding that should be further explored using alternative study designs.

In summary, the present study demonstrated that, in addition to neurons, astrocytes are actively involved in the blood pressure elevation evoked by psychological stress and in particular in the post-stress sustainment of blood pressure elevation. These findings suggest the existence of a causative relationship between astrocytes and hypertension, which targets astrocytes for anti-hypertensive drug development.

## Methods

### Ethics declarations

The study was approved by the Ethics Committee of Murayama Medical Center (approval numbers: 12-1 and 12-2), and was carried out in accordance with the Guiding Principles for the Care and Use of Animals of the Physiological Society of Japan and with Animal Research: Reporting of In Vivo Experiments (ARRIVE) guidelines.

### Cell-type specificity of arundic acid action in cultured astrocytes and neurons

Although arundic acid has been widely used as an astrocyte-specific inhibitory modulator, we conducted experiments to confirm the cell-type specificity of arundic acid actions. We analyzed the inhibitory effects of arundic acid on the excitability of astrocytes and neurons by ratiometric calcium imaging^21,84^. For measurement of intracellular calcium concentration ([Ca^2+^]_i_), primary astrocytes and cerebellar granule neurons were cultured from the cerebral and cerebellar cortices of 0- to 2-day-old C57BL/6 mice, respectively. The primary astrocytes and neurons were separately cultured and loaded with a ratiometric fluorescent calcium indicator, 1 µM Fura-2 AM ester (Dojindo, Kumamoto, Japan), as described in detail^21,84^. The [Ca^2+^]_i_ of cultured astrocytes and neurons was measured by Fura-2 fluorescence ratiometric imaging. The fluorescence was measured at excitation wavelengths of 340 nm and 380 nm (F340 and F380, respectively) and an emission wavelength of 510 nm by a video image analysis system (AQUACOSMOS, Hamamatsu Photonics, Hamamatsu, Japan) at 0.1 Hz sampling frequency. Measurements were conducted following 15 min pre-treatment with the 0.1% DMSO as a vehicle or various (0.01, 0.1 and 1 mM) concentrations of arundic acid with 0.1% DMSO. Maximum changes in [Ca^2+^]_i_ during 10 min stimulation with control (6 mM) or high (55 mM) KCl perfusion solution against 2 min pre-stimulation periods (ΔRatio(F340/F380)) were calculated for the cultured astrocytes and neurons^21,84,85^.

### Cell-type specificity of arundic acid action in acute brain slices

Under deep anesthesia with isoflurane (approximately 0.13%), the brains of 13–15 day-old Wistar rats were rapidly removed and placed in ice-cold artificial cerebrospinal fluid (ACSF) of the following composition (in mM): 118 NaCl, 3 KCl, 1 CaCl_2_, 1 MgCl_2_, 26 NaHCO_3_, 1.2 NaH_2_PO_4_, and 30 glucose, equilibrated with 95% O_2_ and 5% CO_2_, pH 7.4, at 25–26 °C. Transverse medullary slices (500 μm thick) were cut with a vibrating-blade tissue slicer (PR07, DOSAKA EM, Osaka, Japan) and placed in an incubation chamber. A single slice was transferred to a recording chamber, continuously superfused at 2–3 mL/min with the ACSF that was equilibrated with 95% O_2_ and 5% CO_2_, pH 7.4, at 25–26 °C. A total of 10 slices from 5 rats were used for experiments. Arundic acid was stocked as a 1 M in DMSO. A fluorescent calcium indicator Oregon Green 488 BAPTA-1 AM (Oregon Green) (Invitrogen, Carlsbad, CA) was stored as a 200 µM stock solution at −20 °C. To record cellular activities, Oregon Green (200 µM), was pressure-injected into the ventrolateral medulla at the level of the pre-Bötzinger complex. Basic methods for calcium imaging were previously described^86^. In brief, the cell-bound calcium indicator dye was excited by a 488-nm laser and changes in the fluorescence were detected by a Nipkow-disc confocal microscope system (confocal scanner unit: CSU-W1, Yokogawa, Japan; upright microscope: BX51WI, Olympus, Tokyo; objective lens: LUMPLFLN40XW, Olympus; electron-multiplying CCD camera: iXon Ultra, Andor Technology, Belfast, Northern Ireland, UK). The CCD-based camera head has a 13.312 × 13.312 mm imaging area consisting of 1024 × 1024 pixels. The final magnification was ×40 times, so that an area of 0.3328 mm×0.3328 mm was covered by the image sensor. The fluorescence intensity was obtained using live cell imaging software iQ3 (Andor Technology). The fluorescence value of each pixel ranged from 0 to 65,536. The fluorescence was recorded with an exposure time of 200 ms and an acquisition time of 3 Hz for 1000 frames. Mean fluorescence intensity at 20 × 20 pixels of the region of interest was calculated by subtracting values of the adjacent area with 20 × 20 pixels by means of the software Andor SOLIS for Imaging (Oxford Instruments plc, Abingdon, UK). Superfusing ACSF was supplied to the slice preparation via two routes: (1) a main route perfusing the entire recording chamber and a sub-route through a glass tube (inside diameter, 2 mm) that was placed close to the preparation. Under the control condition, the control solution was perfused through both routes. The test solution was applied to the preparation via the sub-route by replacing the control solution with the test solution. The rate of superfusion was set at 2–3 ml/min for each route. This system allowed rapid application (within 30 s) of the test solution to the preparation during measurement of calcium signals for a total of 5 min in one trial^87,88^. Imaged cells were classified into two groups; (1) putative astrocytes based on stronger brightness by Oregon Green staining and smaller cell sizes and (2) putative neurons based on weaker brightness and larger cell sizes^89–91^.

### Immunohistochemical analyses

As preliminary investigation to identify the type of cells that express c-Fos, we first conducted dual staining of (a) c-Fos and a neuron-specific marker NeuN, (b) c-Fos and an astrocyte-specific marker S100 and (c) c-Fos and an astrocyte-specific marker GFAP using cardiovascular brain region tissue of randomly selected rat brains. Then, the main immunohistochemical examinations were conducted in the cardiovascular brain areas CeA, PVN, DMH and RVM as well as in the area that is not involved in cardiovascular regulation, i.e., the rostral ventral medullary area deep from the ventral surface, in 9 rats aged 7 weeks, after they had been bred for 2 weeks under the same condition as blood pressure experiments. The rats were treated under four different conditions. Condition **a**: without arundic acid pre-treatment and without stress loading (Arundic acid −/ Stress −, *n* = 2); Condition **b**: without arundic acid pre-treatment and with stress loading (Arundic acid −/ Stress +, *n* = 3); Condition **c**: with arundic acid pre-treatment and without stress loading (Arundic acid +/ Stress −, *n* = 2); Condition **d**: with arundic acid pre-treatment and with stress loading (Arundic acid +/ Stress +, *n* = 2). The rats under Conditions **c** and **d** received 200 mg/kg arundic acid that was diluted with DMSO akin to the routinely used in the blood pressure experiments. Under Conditions **a** and **b**, without arundic acid injection, DMSO vehicle injection substituted it. Under Conditions **b** and **d**, air-jet stress was loaded in 90 min after injections, but the duration of stress loading was 30 min in the histochemical study followed by further survival for another 30 min. Under Conditions **a** and **c**, rats were kept in cages without stress loading for 150 min after injection of both vehicle and arundic acid. Then, the rats were anesthetized with isoflurane and were transcardially perfused with 0.9% saline followed by 4% paraformaldehyde in 0.1 M phosphate buffer (pH = 7.4). The brain was isolated, post-fixed overnight in the same perfusate at 4 °C, and immersed in 30% cold sucrose. The expressions of cell markers for astrocytes S100 and GFAP^13,92^, for neurons NeuN and for cell activation marker c-Fos^93–95^ were analyzed by double-labeling fluorescent immunohistochemistry together with DAPI staining. The brain samples were cut into 30 μm thick sections on a freezing microtome. The sections were incubated in blocking solution for 30 min, subsequently incubated overnight in blocking solution containing a combination of antibodies of cell markers (S100, S2532, Sigma-Aldrich, 1:500; GFAP, G3893, Sigma-Aldrich, 1:1000; NeuN, MAB377, Millipore/Merck, 1:200) and c-Fos (ABE457, Millipore/Merck, 1:1000). After washed in PBS, the sections were incubated in blocking solution containing Cy3-conjugated anti-rabbit IgG (Jackson Immunoresearch Laboratories, 1:500) and Alexa488-conjugated anti-mouse IgG (Vector Laboratories, Newark, CA, 1:500) with DAPI (FK045, Dojindo, 1:2000) for 3 h. Subsequently, the sections were mounted onto gelatinized slides, coverslipped with antifade mounting medium (Vectashield, Vector Laboratories). Finally, the sections were observed under a fluorescent microscope (BZ-X700; Keyence, Osaka, Japan) with a 40x objective lens, and the expression of c-Fos in the target brain regions was analyzed by triple labeling with antibodies against c-Fos and that against either GFAP or S100 with DAPI staining.

We quantitatively evaluated the degree of astrocytic activation on the basis that the first-order process thickness of astrocytes reflects the level of cell activation^23,24^. We measured the diameters of the first-order processes of GFAP-stained cells in the cardiovascular brain areas CeA, PVN, DMH and RVM at 20 points under each of four different conditions (Conditions **a–d**) using an image analysis software Digimizer (MedCalc Software, Ostend, Belgium).

The CeA can be identified as a round-shaped area located between the corpus callosum and the optic tract. The PVN lies adjacent to the third ventricle. The DMH was defined as the area between the third ventricle and the lateral edge of the mammillothalamic tract^95^. The RVM was defined as the superficial ventral medullary region consisting of the rostral ventrolateral (RVLM) and ventromedial medulla (RVMM)^93^. The RVLM is located ventral to the nucleus ambiguous and caudal to the facial nucleus. The RVMM is located medial to RVLM. Cytoarchitecture of the brain regions was evaluated based on the anatomical atlas^96^.

### Measurement of blood pressure and heart rate

The functional experiments were conducted in male Wistar rats (*n* = 13, aged 5 ~ 9 weeks). The rats were bred under constant room temperature (23–24 °C), 50–60% relative humidity and an alternating 12-hour light/dark cycle with access to standard commercial chow and water *ad libitum*. To measure arterial blood pressure, we applied two methods. In one method (tube catheter method, *n* = 8), a polyethylene catheter (Tombo No.9003 PFA, outer diameter 1.0 mm, Nichias, Tokyo, Japan) was inserted into a unilateral femoral artery under anesthesia with isoflurane followed by pentobarbital sodium injection (40–50 mg/kg, intraperitoneal). The catheter was filled with heparinized saline, and then it was tunneled subcutaneously and exteriorized at the dorsal midline between the scapulae. The catheter tip was covered with a jacket to protect it from biting by the rat. After catheterization, the rats were allowed to recover for at least 48 h. In the other method (telemetric method, *n* = 5), we opened the abdomen under the same anesthetic protocol as above and implanted a wireless telemeter (TRM54P, Kaha Sciences, Auckland, New Zealand) inside the abdominal cavity. We inserted a fine catheter (0.7 mm diameter, 9 cm long) which extended from the main body of the telemeter into the abdominal aorta at the level just rostral to the bifurcation to the common iliac arteries, and the catheter tip was placed at the level of the renal arteries. A solid-state pre-calibrated pressure sensor was equipped at the tip of the catheter, and blood pressure was measured directly at the tip of the catheter. After the telemeter was implanted, the rats were allowed to recover for at least 7 days^77^. In the tube catheter method, the catheter was connected to a pre-calibrated pressure transducer (DX-100, Nihon Kohden, Tokyo, Japan) coupled to a DC amplifier (AP-601G, Nihon Kohden), and arterial blood pressure was continuously measured. In the telemetric method, the pressure signals were continuously transmitted to the signal receiving and processing system (TR181/TR190, Kaha Sciences). In both methods, the raw blood pressure signals were digitized at 200–2000 Hz sampling rates with an A/D converter (PL 3504 PowerLab 4/26, ADInstruments) and stored in PC with LabChart7 software (ADInstruments) for later analysis. From the blood pressure waveforms, MAP was computed, as follows. In each cardiac cycle, the peak and trough values were read out from the waveform as systolic and diastolic pressures, respectively. We calculated MAP as diastolic pressure + 1/3 * (systolic pressure − diastolic pressure). Also, the HR was counted, which precisely coincided with the value obtained by electrocardiographic recording^77^. This computation was conducted by LabChart7 software. Because the tube catheter and the telemetric method yielded practically equal MAP values, we have analyzed the data obtained by these two methods together.

### Loading of stress

To analyze the response of blood pressure to psychological stress, we employed air-jet stress since it induces reproducible excitation of the sympathetic nervous system without hurting animals, i.e., it induces psychological stress but not physical stress^95,97,98^. Briefly, the rat was kept in an animal cage made of transparent plastic (shape cubic, height 20 cm, length 40 cm and width 25 cm) in which it had been domesticated without restraint while the blood pressure was continuously monitored. After the 60 min acclimatization period, resting blood pressure and HR were recorded for 5 min, and the averaged MAP and HR over the 2.5 min period preceding the onset of air-jet stress were defined as the baseline levels. Then, air-jet stress was loaded by blowing compressed air (10 L/min), through a plastic tube with a fine tip (inner diameter of 1.5 mm), toward the animal’s ears for 15 min while recording blood pressure and HR. The animal was allowed to move freely inside the cage during the entire experiment. However, it did not move actively or did not try to escape even during air-jet blowing, and air-jet stress loading could be conducted smoothly. Following the air-jet stress loading, blood pressure and HR in the post-stress recovery phase were recorded for 15 min. Average values of MAP and HR were calculated for 15 min of stress-loading and 15 min of post-stress recovery.

Blood pressure and HR responses to air-jet stress were analyzed before and after intraperitoneal administration of arundic acid, which selectively blocks the activation of astrocytes. We obtained arundic acid from Ono Pharmaceutical Co. (Osaka, Japan) as a generous gift. Arundic acid, which is a clear liquid, was diluted with dimethyl sulfoxide (DMSO) to a final concentration of 225 mg/ml, 1:3 v/v. Although DMSO could affect brain function, the total dose of DMSO used in the present study was lower than that which may affect the autonomic nervous system function^20,99^. Discomfort and pain which could be induced by repeated drug administration were minimized by using of syringes with extremely thin (outer diameter 0.33 mm), so-called pain-free, needles. Blood pressure and HR responses to stress were analyzed before and 60 min after each injection of arundic acid. The sequence of injections was as follows: (1) arundic acid—50 mg/kg, (2) arundic acid—50 mg/kg,—cumulative dose of 100 mg/kg, (3) arundic acid—100 mg/kg,—cumulative dose of 200 mg/kg. The reason why we applied multiple doses arundic acid to the same rats was to analyze the dose-response relationship. The interval time 60 min was chosen on the basis of a previous prestigious report in which an air-jet stress procedure was applied to adult rats to induce psychological stress^97^. Although the pharmacokinetics of arundic acid in in vivo adult rats have not been reported, because the mean terminal half-life of intravenously infused arundic acid in humans is approximately 2 to 3 h^100^, it could be estimated and must take into account that the serum and intra-brain tissue concentrations of arundic acid when a certain dose of arundic acid is administered dividedly as multiple doses would be lower than those when arundic acid is administered as a single high dose.

### Statistical analysis

The diameters of the first-order astrocytic processes were compared across four brain regions (CeA, PVN, DMH, RVL) under four different conditions, defined by the presence or absence of arundic acid (Arundic acid (+), Arundic acid (−)) and the presence or absence of stress (Stress (+), Stress (−)); Groups 1–4. Welch’s t-test was employed to assess statistical differences between the groups. To correct for multiple comparisons within each brain region, the Bonferroni method was applied. As there are six pairwise comparisons within each region, the significance level for each t-test was adjusted to 0.05/6.

For MAP and HR, averaged mean baseline values (over the 2.5 min preceding stress loading) were compared with a one-way analysis of variance (ANOVA) between the drug dose factors. Then, mean increase values relative to the baseline of MAP and HR (ΔMAP and ΔHR) during 15 min stress-loading and 15 min post-stress recovery were compared respectively by the Welch’s t-test in 13 rats. The Bonferroni correction was employed to adjust p-values for the multiple-test comparisons. Data are presented as means ± SE. The significance of changes was set at the alpha level of *p* < 0.05.

## Electronic supplementary material

Below is the link to the electronic supplementary material.

Supplementary Material 1

## Data Availability

The data analyzed in the current study are available from the corresponding author upon reasonable request.
