# Supplementary material for: Persistence of post-stress blood pressure elevation requires activation of astrocytes
Source: Sci Rep. 2024 Oct 3;14:22984. doi: 10.1038/s41598-024-73345-4 (PMC11450218; doi:10.1038/s41598-024-73345-4)
Supplement: Supplementary file 1 — Supplementary Material 1 [file 41598_2024_73345_MOESM1_ESM.pdf]

# Supplementary information

## Persistence of post-stress blood pressure elevation requires activation of astrocytes

Yohei Hasebe[1,2], Shigefumi Yokota[3], Isato Fukushi[2,4], Kotaro Takeda[2,5], Hiroshi Onimaru[6], Masashi Yoshizawa[1,2], Yosuke Kono[1,2], Shuei Sugama[7], Makoto Uchiyama[8], Keiichi Koizumi[1], Jouji Horiuchi[9], Yoshihiko Kakinuma[10], Mieczyslaw Pokorski[11], Takako Toda[1], Masahiko Izumizaki[6], Yasuo Mori[8], Kanji Sugita[1], Yasumasa Okada[2*]

[1] Department of Pediatrics, School of Medicine, University of Yamanashi, Chuo, Yamanashi, Japan

[2] Clinical Research Center, Murayama Medical Center, Musashimurayama, Tokyo, Japan

[3] Department of Anatomy and Morphological Neuroscience, Shimane University School of Medicine, Izumo, Shimane, Japan

[4] Faculty of Health Sciences, Aomori University of Health and Welfare, Aomori, Japan

[5] Faculty of Rehabilitation, School of Health Sciences, Fujita Health University, Toyoake, Aichi, Japan

[6] Department of Physiology, Showa University. School of Medicine, Tokyo, Japan

[7] Center for Medical Sciences, International University of Health and Welfare, Otawara, Tochigi, Japan

[8] Department of Synthetic Chemistry and Biological Chemistry Graduate School of Engineering, Kyoto University, Kyoto, Japan

[9] Department of Biomedical Engineering, Graduate School of Science & Engineering, Toyo University, Saitama, Japan

[10] Department of Physiology, Nippon Medical School, Tokyo, Japan

[11] Institute of Health Sciences, University of Opole, Opole, Poland

# Figure S1

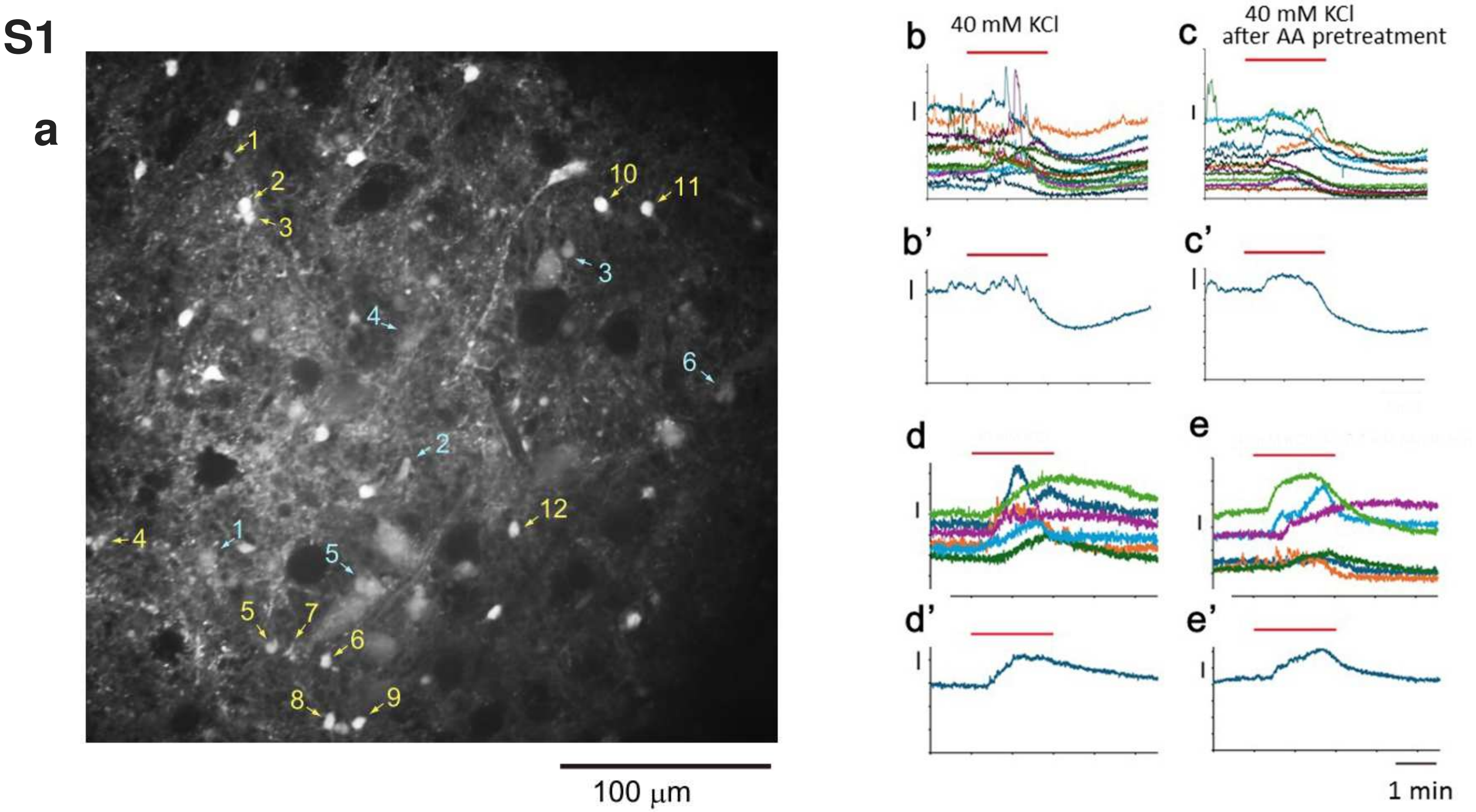


**A typical example of arundic acid (AA) action on cellular responses to high K+. (a)** Optical image of the cut surface of the rostral ventrolateral medulla at the level of the pre-Bötzinger complex stained with Oregon Green. Imaged cells were classified into two groups based on their cell sizes and brightness; putative astrocytes (No. 1-12 with yellow numbering) and putative neurons (No. 1-6 with light blue numbering). **(b)** and **(d)** Changes of fluorescence intensity in response to 40 mM KCl in the control condition (without arundic acid) of putative astrocytes and neurons, respectively. **(c)** and **(e)** Responses to 40 mM KCl after 30 min pretreatment with 0.1 mM arundic acid of putative astrocytes and neurons, respectively. Spike-like calcium increases induced by high K+ in putative astrocytes (b) were eliminated by pretreatment with arundic acid (c). **(b')**, **(c')**, **(d')** and **(e')** Averaged fluorescence intensities of (b), (c), (d) and (e), respectively. Vertical bars on the left of each graph denote 20% change in fluorescence intensity (ΔF/F).

## Figure S2

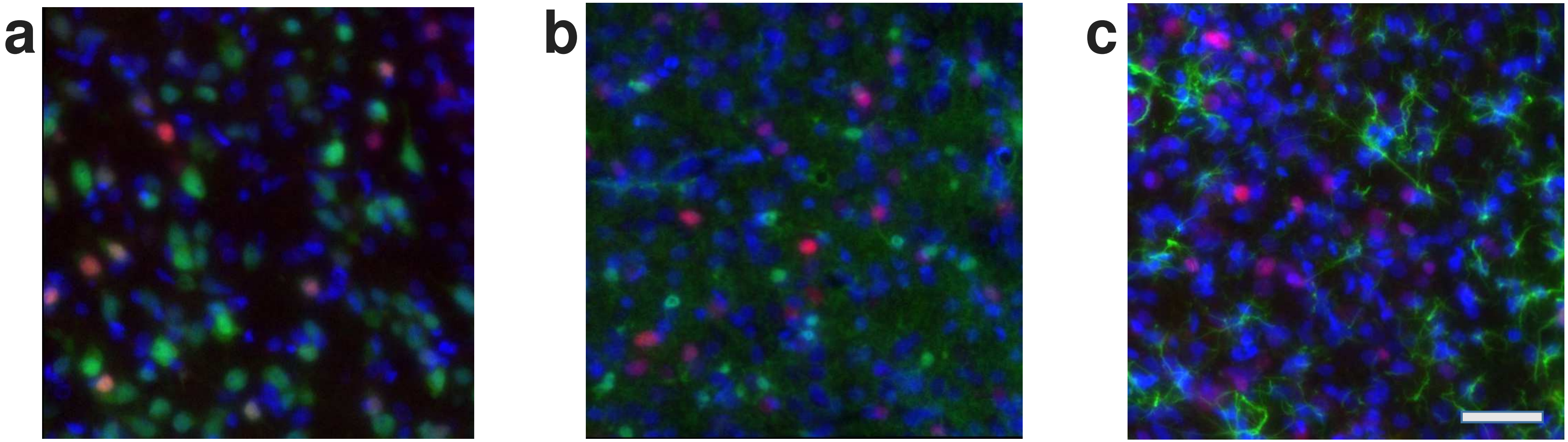

**Cell type specificity of c-Fos positive cells**. **(a)** Triple staining for c-Fos (red), NeuN (green) and DAPI (blue) in the PVN. Most of the c-Fos positive cells were also NeuN positive (orange color), indicating that most of c-Fos positive cells were neurons. **(b)** Triple staining for c-Fos (red), S100 (green) and DAPI (blue) in the PVN. Most of the c-Fos positive cells were S100 negative (not orange but magenta in color), indicating that most of the c-Fos positive cells were not astrocytes. **(c)** Triple staining for c-Fos (red), GFAP (green) and DAPI (blue) in the PVN. Most of the c-Fos positive cells were GFAP negative, indicating that most of the c-Fos positive cells were not astrocytes. Collectively, these findings indicate that most of c-Fos positive cells were neurons. Scale bar, 30 µm.

## Figure S3

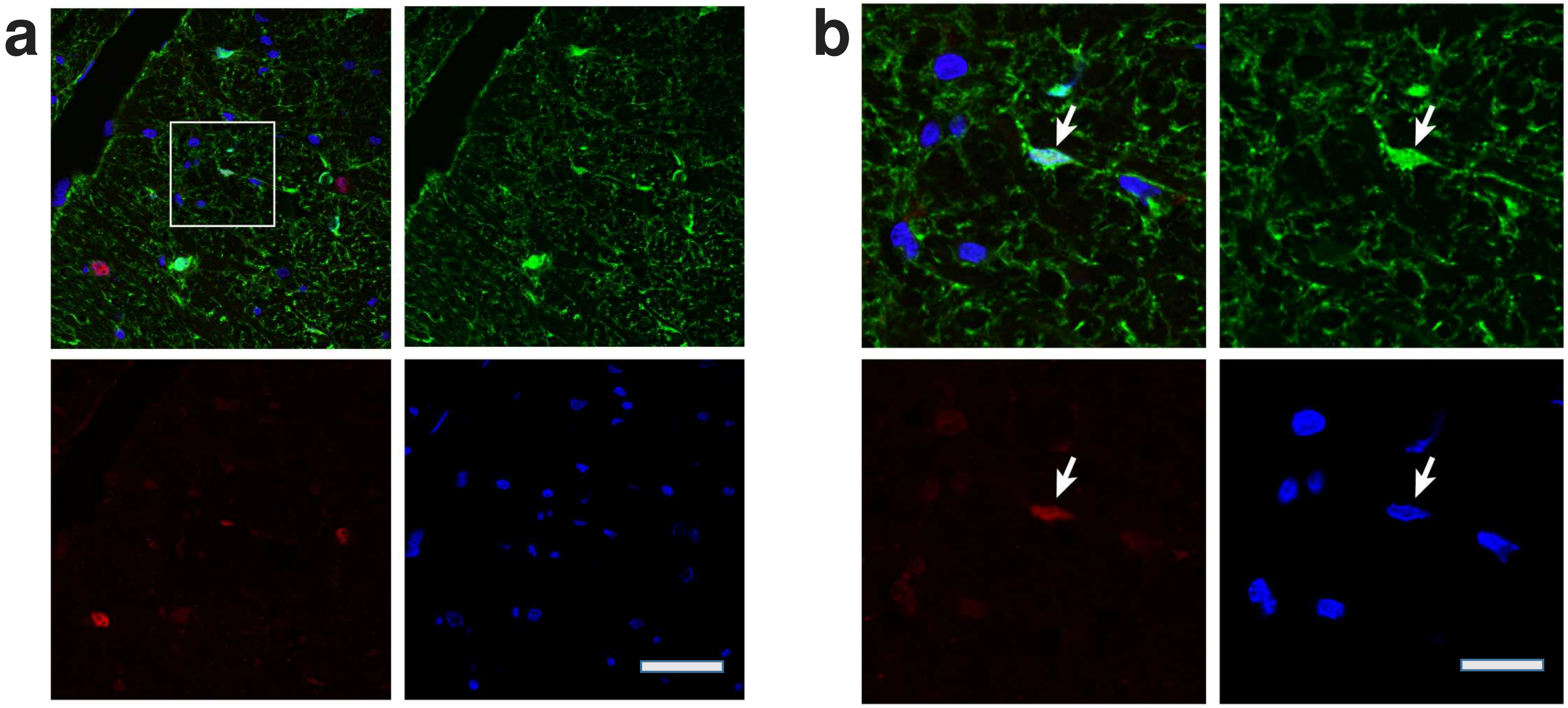


**A representative sample cell with dual positivity for c-Fos (red) and S100 (green).** Although it was rare, there were cells dual positive for c-Fos and S100, e.g. the cell with an arrow, indicating that astrocytes could express c-Fos presumably when they are strongly stimulated. Blue, DAPI stained cell nuclei. Scale bars, **a,** 60 µm, **b,** 20 µm.

| | |
|---|---|
| Merge | S100 |
| c-Fos | DAPI |

## Figure S4

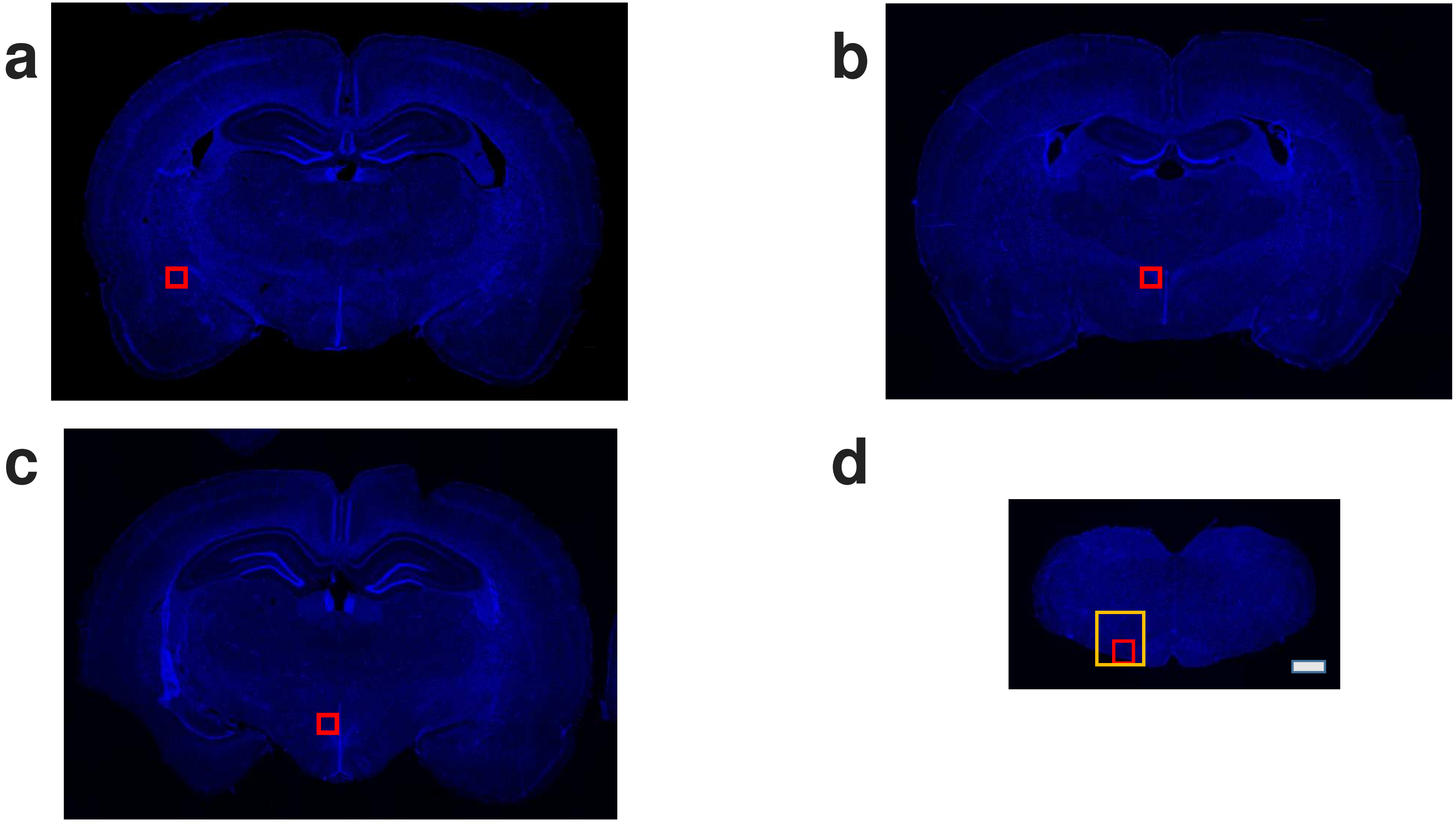


**Locations of the regions selected for immunohistological images.** The regions are shown on DAPI stained brain section pictures. **(a)** The location of the CeA region shown in Figs. 2 and 6. **(b)** The PVN region shown in Figs. 3 and 7. **(c)** The DMH region shown in Figs 4 and 8. **(d)** The RVM region shown in Figs. 5 and 9 (red square) and the non-cardiovascular medullary region deep from the ventral surface shown in Figs. S6 and S7 (orange square). Upward; dorsal. Scale bar, 1 mm.

## Figure S5

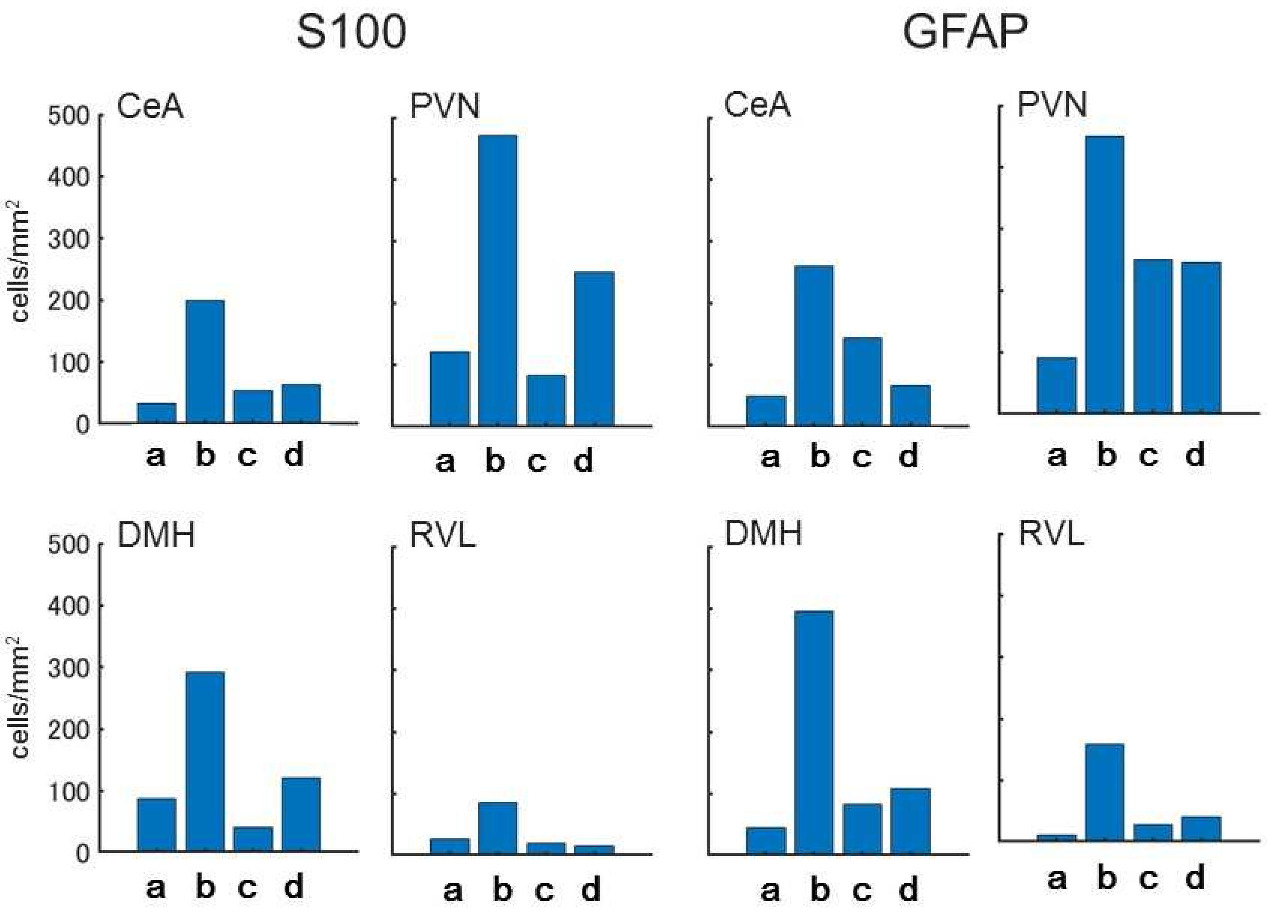


**Densities of c-Fos positive cells in the conditions co-stained with S100 and GFAP in four cardiovascular brain regions (CeA, PVN, DMH, RVL). (a)** Condition **a** (Group1): without arundic acid pre-treatment and without stress loading; **(b)** Condition **b** (Group 2): without arundic acid pre-treatment and with stress loading; **(c)** Condition **c** (Group 3): with arundic acid pre-treatment and without stress loading; **(d)** Condition **d** (Group 4): with arundic acid pre-treatment and with stress loading. To quantitatively demonstrate the c-Fos immunostaining results, the number of c-Fos positive cells was manually counted, and the c-Fos positive cell densities were calculated and graphically presented. Because the differences were clear (the c-Fos positive cell density in Group 2 was much higher than others) and the number of rats analyzed was small, we did not dare to conduct a statistical analysis of the counted results.

## Figure S6

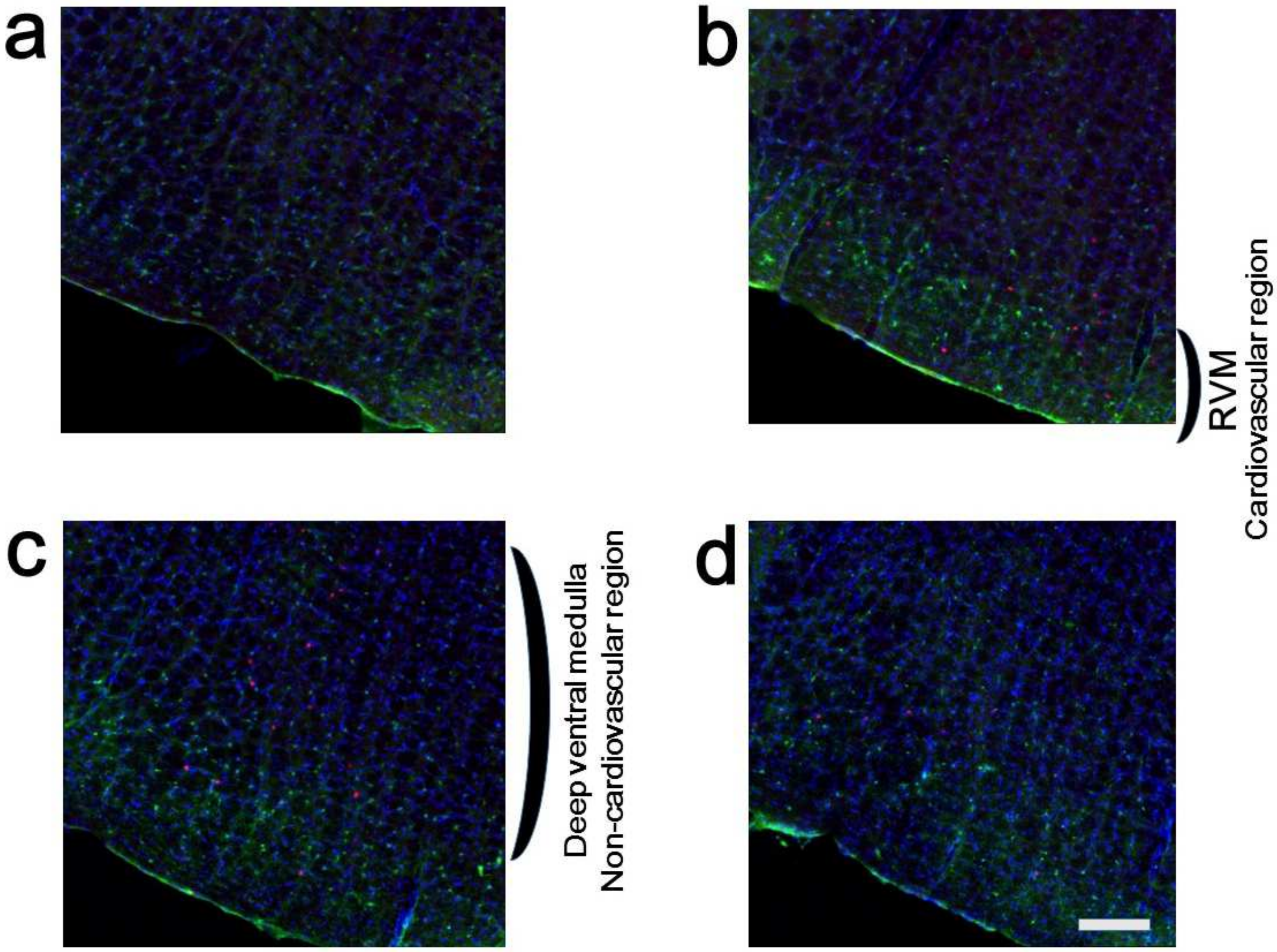


**Double fluorescent immunostaining for c-Fos** (red) **and S100** (green) **with DAPI (blue) staining in the non-cardiovascular region (rostral medulla deep from the ventral surface). (a)** Condition **a** without arundic acid pre-treatment and without stress loading; **(b)** Condition **b**: without arundic acid pre-treatment and with stress loading; **(c)** Condition **c**: with arundic acid pre-treatment and without stress loading; **(d)** Condition **d**: with arundic acid pre-treatment and with stress loading. In Condition **b** a number of c-Fos positive cells were observed in the superficial ventral medullary region, i.e., in the cardiovascular region RVM. In Condition **c** scattered c-Fos positive cells were observed in the deep ventral medullary region, i.e., in the non-cardiovascular region. Bottom oblique line, ventral medullary surface. Scale bar; 150 μm.

# Figure S7

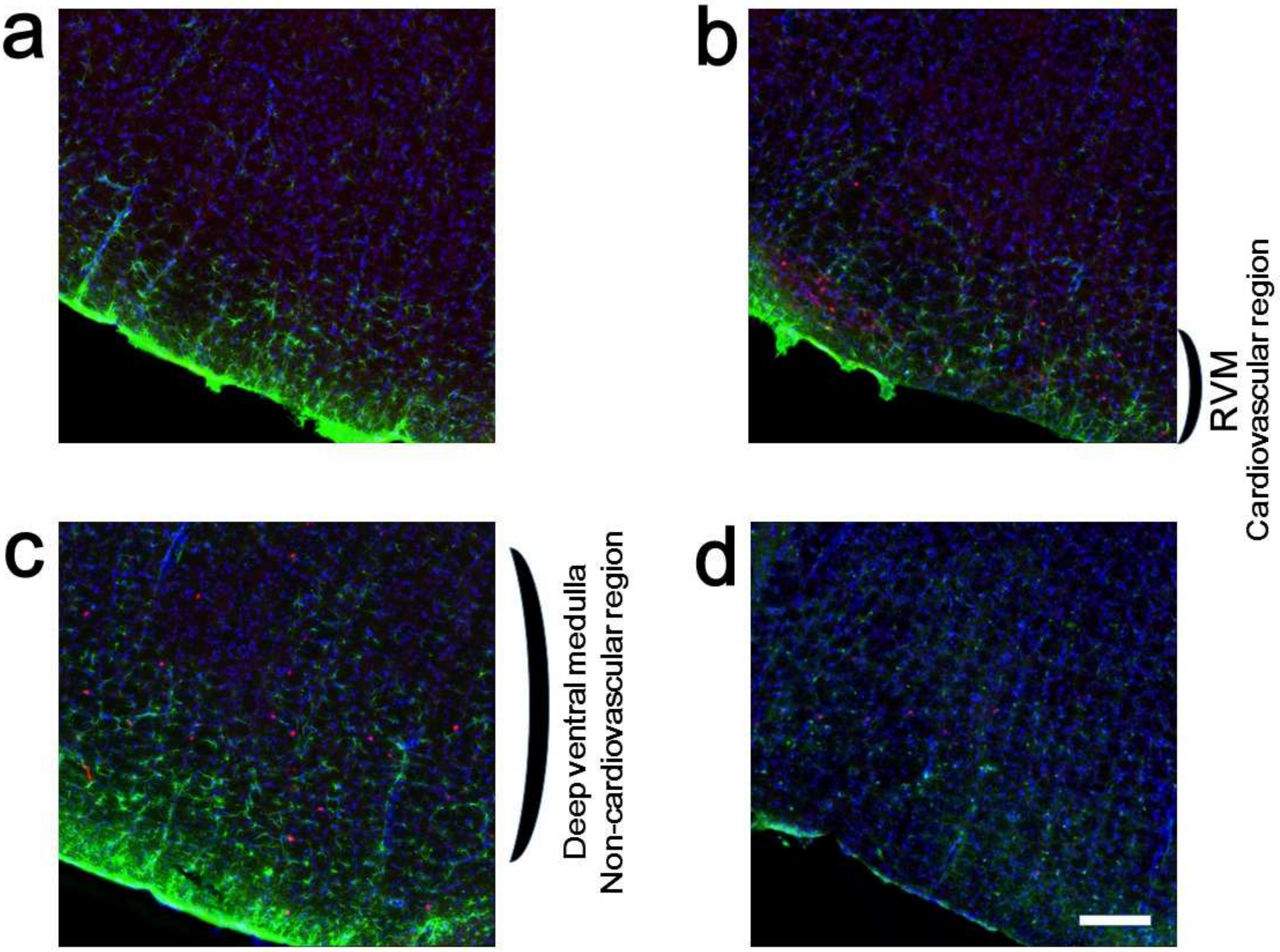


**Double fluorescent immunostaining for c-Fos (red) and GFAP (green) with DAPI (blue) staining in the non-cardiovascular region (rostral medulla deep from the ventral surface). (a)** Condition **a** without arundic acid pre-treatment and without stress loading; **(b)** Condition **b**: without arundic acid pre-treatment and with stress loading; **(c)** Condition **c**: with arundic acid pre-treatment and without stress loading; **(d)** Condition **d**: with arundic acid pre-treatment and with stress loading. In Condition **b** a number of c-Fos positive cells were observed in the superficial ventral medullary region, i.e., in the cardiovascular region RVM. In Condition **c** scattered c-Fos positive cells were observed in the deep ventral medullary region, i.e., in the non-cardiovascular region. Bottom oblique line, ventral medullary surface. Scale bar; 150 μm.

# Figure S8

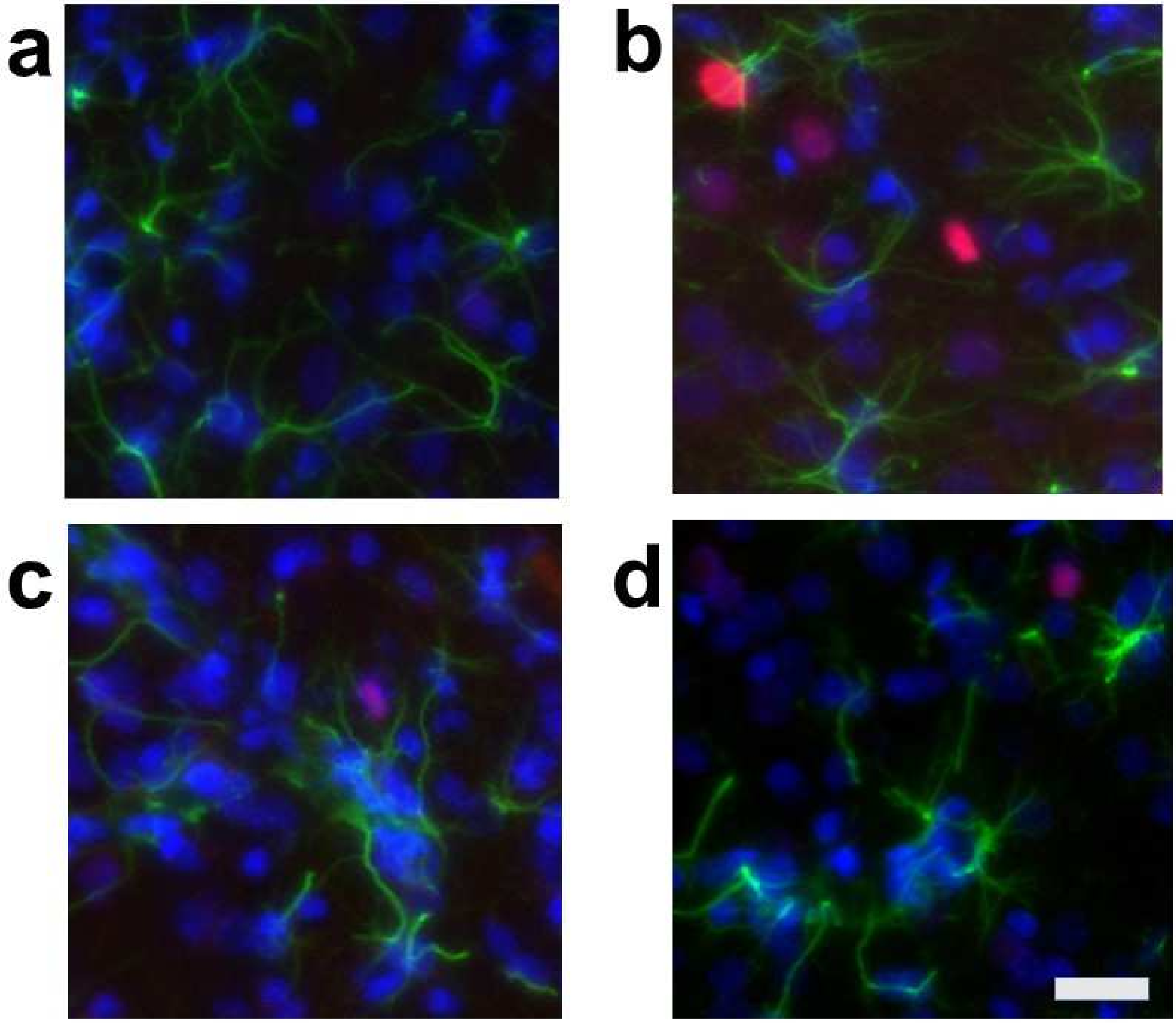


**Comparison of process thicknesses of GFAP-stained putative astrocytes (green) among different conditions in the CeA. (a)** Condition **a** without arundic acid pre-treatment and without stress loading; **(b)** Condition **b**: without arundic acid pre-treatment and with stress loading; **(c)** Condition **c**: with arundic acid pre-treatment and without stress loading; **(d)** Condition **d**: with arundic acid pre-treatment and with stress loading. In Condition **b** the first-order processes were thicker than those in other conditions. Blue: DAPI. Red: c-Fos. Scale bar; 20 μm.
